# TOP-PIC: a new tool to optimize pharmacotherapy and reduce polypharmacy in patients with incurable cancer

**DOI:** 10.1007/s00432-023-04671-9

**Published:** 2023-03-06

**Authors:** Irene Strassl, Armin Windhager, Sigrid Machherndl-Spandl, Veronika Buxhofer-Ausch, Olga Stiefel, Ansgar Weltermann

**Affiliations:** 1 Division of Hematology With Stem Cell Transplantation, Hemostaseology and Medical Oncology, Department of Internal Medicine I, Ordensklinikum Linz, Fadingerstrasse 1, 4020 Linz and Seilerstätte 4, 4010 Linz, Austria; 2 Doctoral Programme MedUni Vienna, Medical University of Vienna, Spitalgasse 23, 1090 Vienna, Austria; 3 Medical Faculty, Johannes Kepler University Linz, Altenberger Strasse 69, 4040 Linz, Austria; 4 Department of Cardiology and Intensive Care Medicine, Kepler University Hospital Linz, Krankenhausstrasse 9, 4021 Linz, Austria

**Keywords:** Polypharmacy, Cancer, Clinical decision-making, Risk assessment, Drug therapy, Potentially inappropriate medication list

## Abstract

**Purpose:**

Polypharmacy is a significant problem in patients with incurable cancer and a method to optimize pharmacotherapy in this patient group is lacking. Therefore, a drug optimization tool was developed and tested in a pilot test.

**Methods:**

A multidisciplinary team of health professionals developed a “Tool to Optimize Pharmacotherapy in Patients with Incurable Cancer” (TOP-PIC) for patients with a limited life expectancy. The tool consists of five sequential steps to optimize medications, including medication history, screening for medication appropriateness and drug interactions, a benefit–risk assessment using the TOP-PIC Disease-based list, and shared decision-making with the patient. For pilot testing of the tool, 8 patient cases with polypharmacy were analyzed by 11 oncologists before and after training with the TOP-PIC tool.

**Results:**

TOP-PIC was considered helpful by all oncologists during the pilot test. The median additional time required to administer the tool was 2 min per patient (*P* < 0.001). For 17.4% of all medications, different decisions were made by using TOP-PIC. Among possible treatment decisions (discontinuation, reduction, increase, replacement, or addition of a drug), discontinuation of medications was the most common. Without TOP-PIC, physicians were uncertain in 9.3% of medication changes, compared with only 4.8% after using TOP-PIC (*P* = 0.001). The TOP-PIC Disease-based list was considered helpful by 94.5% of oncologists.

**Conclusions:**

TOP-PIC provides a detailed, disease-based benefit–risk assessment with recommendations specific for cancer patients with limited life expectancy. Based on the results of the pilot study, the tool seems practicable for day-to-day clinical decision-making and provides evidence-based facts to optimize pharmacotherapy.

**Supplementary Information:**

The online version contains supplementary material available at 10.1007/s00432-023-04671-9.

## Background

Polypharmacy is a common medical problem as chronic health conditions are increasing in the older population and one or more medicines may be used to treat each condition (Bennett and Sofat 2020). In a European study of persons 65 years or older, polypharmacy prevalence ranged from 26 to 40% (Midão et al. 2018). The definition of polypharmacy is challenging as the term is not clearly defined and more than 100 different definitions exist (Masnoon et al. 2017). The intake of five or more medications daily is the most commonly used definition as this number was shown to be a risk factor for severe drug interactions and adverse drug reactions (Lau et al. 2005). In addition to the numerical definition of polypharmacy, the assessment of safety and appropriateness of therapy is essential. In a review of 110 articles with 138 polypharmacy definitions, only 6% of articles classified inappropriate polypharmacy and 80% utilized numerical definitions only (Masnoon et al. 2017).

The use of multiple medications is steadily increasing among the geriatric population (Payne 2016). As polypharmacy is a predictor of hospitalizations, nursing home placement and death in older adults (Davies et al. 2020; Turgeon et al. 2017; Wastesson et al. 2018a, b), extensive research has been carried out resulting in a number of criteria or tools to reduce polypharmacy, drug–drug interactions (DDIs) and potentially inappropriate medications (PIMs) (de Agustín Sierra et al. 2021; Niehoff et al. 2019; Wastesson et al. 2018a, b). Existing tools that assist doctors to select appropriate drugs for the geriatric population show clear benefits, including less adverse drug reactions (ADRs), fewer and shorter hospitalizations and an improvement in activities of daily living (Hill-Taylor et al. 2016; Wehling et al. 2016). Most existing criteria to reduce polypharmacy are primarily derived from the general geriatric population. Yet polypharmacy, ADRs, PIMs and DDIs are also frequently seen in patients with cancer (Ramsdale et al. 2022; Sharma et al. 2016), but there is a lack of practicable deprescribing tools for these patients. Data regarding the impact of polypharmacy and PIMs on outcomes in oncologic patients are limited and study results are inconsistent. This may be due to the heterogeneity of definitions and populations. Furthermore, older patients and patients with cancer are often underrepresented in clinical trials addressing polypharmacy. Different studies in patients with cancer showed associations of polypharmacy or DDIs with postoperative complications, chemotherapy toxicity, unexpected hospitalizations, and mortality (Chen et al. 2020; Hakozaki et al. 2020, 2021; Hong et al. 2020; Lu-Yao et al. 2020; Mohamed et al. 2020), while other trials could not confirm these associations, especially regarding mortality (Beinse et al. 2020; Karuturi et al. 2019; Mohamed et al. 2020). Unplanned or more frequent hospitalizations were most often associated with polypharmacy (Hakozaki et al. 2020; Hong et al. 2020; Lu-Yao et al. 2020). Polypharmacy also plays a major role in the occurrence of delirium-related hospital admission or prolonged hospitalization in cancer patients (Lawlor & Bush 2015; Şenel et al. 2017). Thus, the relevance of a drug optimization tool in cancer patients is given. The two most obvious problems in the utilization of geriatric tools for patients with cancer are that many prescribed supportive care medications, such as analgesics or antiemetics, are considered potentially inappropriate in these tools, and that the limited life expectancy of patients with metastatic disease is not considered (Mohamed et al. 2020; Zhan et al. 2001). If geriatric medication screening tools were used in older patients with cancer, a high number of PIMs of up to 73% were identified in several studies (Hong et al. 2020; Kotlinska-Lemieszek et al. 2014; Lavan et al. 2021; Whitman et al. 2018). The prevalence of potential DDIs in patients with cancer receiving antineoplastic therapy ranged from 24 to 98% (Castro-Manzanares et al. 2019; Hoemme et al. 2019; Ismail et al. 2020; Nightingale et al. 2018; Prely et al. 2021; Ramasubbu et al. 2021).

Taking into account the high prevalence of polypharmacy, PIMs and DDIs in patients with cancer (Kotlinska-Lemieszek et al. 2014), deprescribing medications becomes relevant, especially when life expectancy decreases due to metastatic disease. Previously, appropriate therapies have to be re-examined, because preventive medications may no longer be beneficial. Typical drugs are lipid-lowering drugs, antihypertensive drugs, osteoporosis drugs and hyperglycemic drugs. Most studies that have addressed discontinuation of these drugs in patients with a limited life expectancy found it to be safe and feasible (Brokaar et al. 2021; Haider et al. 2020). Evidence regarding quality of life and other outcomes after deprescribing preventive drugs is sparse (Brokaar et al. 2021). As tools to reduce polypharmacy and optimize medications for the oncologic population are rare, developing a tool to optimize pharmacotherapy in patients with incurable cancer and a limited life expectancy was needed to fill this gap.

## Materials and methods

### Intent of the TOP-PIC tool and development process

The TOP-PIC tool is intended for use in all cancer patients with incurable disease and a limited life expectancy of approximately three years or less. It has no age restrictions in comparison with many other tools, and it was not developed for palliative patients with a noticeably short life expectancy of a few weeks. The tool assists clinicians to identify PIMs and DDIs, to add missing medications for given indications, and to decide which medication is not necessary anymore because of the decreased life expectancy. The goal of the TOP-PIC tool is to reduce PIMs, DDIs and ADRs, and thus to improve the quality of life and tolerability of cancer therapy in this patient group. As prescribing decisions are never easy and clinicians have to consider multiple factors, the tool provides a list of recommendations for the attending oncologist to enable faster, more evidence-based decision-making as part of the clinical routine.

**Fig. 1:**
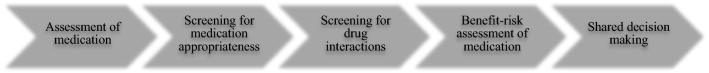
TOP-PIC: schematic description of the five-step process

The development of the TOP-PIC tool started with a review of the existing literature. We searched for polypharmacy in general, as well as existing tools for prescribing and deprescribing in the geriatric and the oncologic population, tools to evaluate drug interactions and adverse drug reactions, and outcomes of patients with polypharmacy and outcomes after usage of different existing tools. Based on the findings of the literature search, we developed an approach consisting of five sequential steps we consider essential in reducing polypharmacy and increasing patient adherence to treatment. The optimization process begins with a precise assessment of all medications and comorbidities (1st step), is followed by a screening for medication appropriateness (2nd step), drug-drug interactions (3rd step), a benefit-risk assessment (4th step), and ends with a discussion and a shared decision together with the patient (5th step) (Fig. 1). TOP-PIC is intended to be used by the treating oncologist who knows not only the current medications, but also the past and current general condition, comorbidities, the scheduled oncological treatment and the preferences of the patient.

1. Assessment of medication and comorbidities

The first step to optimize the medication of a patient is the precise assessment of all current drugs and comorbidities. In addition to the anamnesis questionnaire, a discussion of all medications is important right at the start because patients often tend to forget to name drugs they receive from other doctors or over the counter. It is also necessary to record not only the long-term medication, but also drugs that are taken on demand or in intervals. Furthermore, patients often take complementary or herbal drugs and it is relevant to ask about such medications. After taking a detailed medical history, including proactively inquiring about all medications taken, all drugs are documented on a printed form. The patient has to fill in the name of the drug, the dosage, the route and frequency of application. In addition, information on side effects, compliance, and willingness to stop or maintain a medication should be provided. This additional information will be incorporated into the shared decision process in the fifth step. All medications are recorded in a database to enable quick access and to recognize medication changes at follow-up visits.

2. Screening for medication appropriateness

**Fig. 2:**
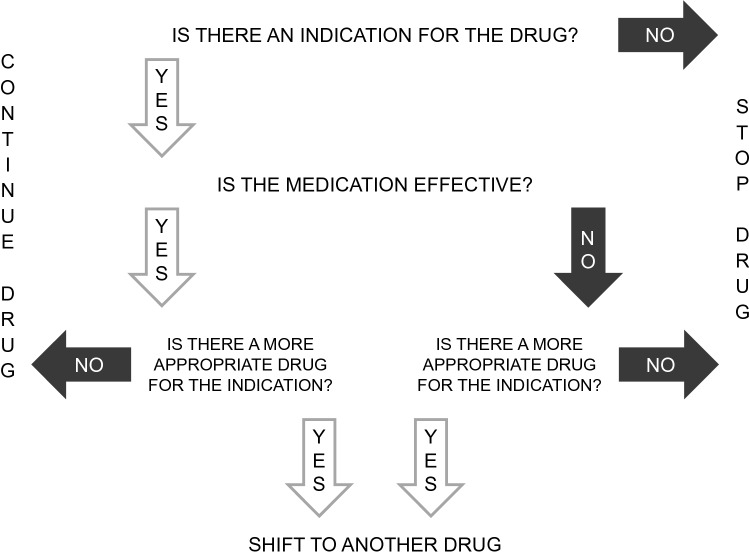
Decision diagram: screening for medication appropriateness

After assessment of all medications, each drug is screened for appropriateness. For this screening process, we use an algorithm modified from the Medication Appropriateness Index (MAI) and the Good Palliative-Geriatric Practice Algorithm (Fig. 2) (Garfinkel et al. 2007; Hanlon et al. 1992). Each drug is screened for indication, effectiveness, and duplication. If a more appropriate drug is available, a shift to another drug should be considered. Missing drugs with a given indication are added.

3. Screening for drug interactions

DDIs and the risk for ADRs are determined with the computer-based drug-interaction tool Diagnosia^®^. The medication can be entered in an online database that includes all approved medications in Austria. The medical database for a DDI check and risk assessment in Diagnosia^®^ has been derived from Medbase^®^ (www.medbase.fi). Data are produced by experienced consultants in clinical pharmacology and therapeutics and further developed by an international team of experts including those from Karolinska Institutet in Sweden. Data are fully referenced and always up-to-date. The concept of risk assessment and clinical experience with Medbase^®^ have been published (Andersson et al. 2013; Böttiger et al. 2009). According to the Medbase^®^ database, DDIs are classified into four categories from A to D and an evidence level from 0 to 4 (Table 1). A risk-score shows the probability of adverse drug reactions in different categories, for example bleeding risk, renal toxicity, seizures, constipation or long QT syndrome.

**Table 1:** Classification of drug–drug interactions adapted from Diagnosia^®^

Risk for interactions		Evidence level	
A	Minor interaction without clinical relevance	0	Data from extrapolation based on studies with similar drugs
B	Clinical consequences are uncertain and/or can vary	1	Data from fragmentary case reports and/or in vitro studies
C	Clinically relevant interaction, which can be regulated with a dose-adaptation for example	2	Data from well documented case reports
D	Clinically relevant interaction, which should be avoided	3	Data from studies with healthy volunteers and/or pilot-studies with patients
		4	Data from controlled trials with a relevant patient population

4. TOP-PIC Disease-based list for benefit–risk assessment of medication

For this important step, we developed the “TOP-PIC Disease-based list” consisting of a comprehensive list of 90 common diseases, their corresponding ICD-10 code (World Health Organization. (2015). International statistical classification of diseases and related health problems), recommendations for patients with a limited life expectancy due to incurable cancer, and further information such as the number needed to treat (NNT) to prevent different outcomes and general recommendations for specific drugs. An excerpt from the list can be found in Table 2 and the complete list in Table S1 (Online Resource 1). The information originates from guidelines of the specialist societies, other published guidelines for particular diseases, and UpToDate^®^. Certain recommendations were adjusted to consider the limited life expectancy of the patient group, especially if a benefit of a drug is expected to occur after many years and has no short-term advantages. The TOP-PIC Disease-based list was primarily compiled by a small team of oncologists, then sent to one to three specialists in the relevant fields and adapted again according to their recommendations. Clinical pharmacologists reviewed the entire list and provided suggestions for improvement. In the end, the list was finalized based on consensus with 32 experts including specialists from internal medicine, pneumology, surgery, dermatology, urology, clinical microbiology, nuclear medicine, psychiatry and clinical pharmacology. In contrast to existing tools, our tool gives recommendations for different diseases instead of medications, which may be more practical for the treating physician. The treating oncologist should use the list to perform a benefit–risk assessment for each individual patient. The list was developed to provide easily accessible information in clinical routine to improve deprescribing and prescribing decisions. As it is not possible to make general recommendations or rules for all patients, an experienced oncologist should always be part of the decision-making process.

**Table 2:** Excerpt from the TOP-PIC Disease-based list for benefit–risk assessment of medications for cancer patients with limited life expectancy (see table S1 for full list)

Specialty	Disease	Recommendation for cancer patients with limited life expectancy	Additional information/comment
Cardiology	Acute coronary syndrome within the last year (underlying coronary artery disease)	Dual antiplatelet therapy after ACS according to cardiological recommendation (PPI recommended) Beta-blockers: continuous therapy indicated after cardiogenic shock or additional cardiac or renal insufficiency Statin: 80 mg atorvastatin or 20 to 40 mg rosuvastatin Antianginal therapy: see confirmed atherosclerotic cardiovascular disease	Benefit of aspirin given for 5 years in preventing a new vascular event: Patients with ACS: NNT 20 in 6 months Patients after myocardial infarction, stroke or TIA: NNT 25 after 2–3 years Benefits of statins given for 5 years for secondary prevention in patients with CVD NNT 83 to save one life NNT 39 to prevent one non-fatal heart attack NNT 125 to prevent one stroke
Endocrinology	Diabetes mellitus type-2	Therapy goal: HbA1c < 8 mg% (strive for stricter goal in case of manifest secondary diseases), avoidance of hypoglycemia Maintain therapy regime if the patient is well adjusted Close monitoring of blood glucose levels during chemotherapy/cortisone Oral therapy only: attempt to reduce dosage Combined therapy with insulin plus oral agents: aim for the simplest possible therapy regime	Higher HbA1c values may be accepted after individual consideration
Orthopedics	Arthrosis (polyarthrosis, coxarthrosis, gonarthrosis, arthrosis of the spinal column, etc.), spondylosis	Maintain or initiate non-pharmacological therapy (physiotherapy/physical activity) Therapy attempt with topical NSAIDs possible (exceptions: polyarthrosis, coxarthrosis); capsaicin patches Oral NSAIDs at the lowest possible dose, combination with PPI in patients with comorbidities such as diabetes, hypertension or advanced age Opiates and paracetamol often not very effective (therapy trial possible, discontinue if ineffective) Discontinuation of food supplements such as glucosamine, chondroitin, vitamin D, diacerein, avocado soybean oil and fish oil	Pain reduction through topical diclofenac (especially in gonarthrosis): NNT 9.8 Pain reduction and improvement of mobility through physical training in coxarthrosis: NNT 6
Urology	Benign prostate hyperplasia	Therapy only in case of obstruction-related, disturbed bladder emptying or restriction of quality of life; alpha-1 blockers are the therapy of choice; 5-alpha-reductase inhibitors in case of intolerance (onset of action after 6–12 months); anticholinergics in case of irritative symptoms (hyperactive bladder); phosphodiesterase-5 inhibitors in case of additionally existing erectile dysfunction; no phytotherapeutics	Alpha-1 blocker vs placebo within 4 years: NNT 19 (3 years) to avoid clinical progression Finasteride vs placebo: surgery for BPH 5% VS 10%- > NNT 20 (4 years)- > NNT 27 (3 years) to avoid surgery; NNT 33 (3 years) for prevention of acute urinary retention

*ACS*  acute coronary syndrome, *BPH*  benign prostatic hyperplasia, *CVD* cardiovascular disease, *NNT*  number needed to treat, *NSAID*  non-steroidal anti-inflammatory drug, *PPI* proton pump inhibitor, *TIA* transient ischemic attack

5. Shared decision-making

After assessment, screening processes and modification of medications, the final medication list should be discussed with the patient, explaining potential benefits and risks of stopping or adding different drugs. The result of the shared decision-making is documented in the patient file and for the primary care physician.

### Practicability evaluation

To evaluate the applicability of the TOP-PIC tool, a pilot test was conducted. For this purpose, 8 patient cases each were processed by 11 experienced oncologists, once without further assistance and once after appropriate training using TOP-PIC. The patient cases consisted of patients with metastatic cancer who were affected by polypharmacy, taken from our retrospective database. Every patient took at least five drugs permanently. Since the patient cases were retrospective, only steps 2 through 4 of the TOP-PIC tool were performed. As a substitute for the first step, the oncologists received the patient history, comorbidities, and medication list. With the information available, they had to decide whether and which medications to change (no change, discontinuation, reduction, increase, replacement, or addition of a drug). For each decision, the certainty of the decision had to be indicated. In the second part of the survey, additional questions were asked about which step of the TOP-PIC tool (steps 2 to 4) was used to make decisions and whether TOP-PIC was helpful or not.

In addition to processing the patient cases, the physicians also answered questions on how to deal with polypharmacy in everyday life.

### Statistical analysis

Statistical analyses were performed using IBM SPSS Statistics for Macintosh, Version 28.0 (Armonk, NY: IBM Corp). Descriptive statistics were used to evaluate the baseline characteristics of the patient cases and physicians as well as the questionnaires on polypharmacy. Continuous data including years of professional experience, time spent on anamnesis and time needed to process the patient cases were summarized as the median (range). The McNemar test was used to examine whether a change occurred in medication and decision confidence in Part 1 versus Part 2, the Wilcoxon signed rank test for comparison of continuous variables. *P* values < 0.05 were considered statistically significant.

## Results

Eleven experienced oncologists evaluated 8 patient cases each with a total of 1065 treatment decisions. See Table 3 for baseline characteristics of patient cases.

**Table 3:** Baseline characteristics of patient cases (*n* = 8)

Median age, years (range)	72.5 (58–84)
Comorbidities^a^, *n* (range)	4 (2–16)
Medication^b^, *n* (range)	12 (8–16)
Permanent medication, *n* (range)	10 (7–12)
Medication on demand, *n* (range)	1 (1–3)
Cancer type, *n* (%)	
Colorectal cancer	3 (38)
Breast cancer	3 (38)
Esophageal cancer	1 (13)
Cholangiocellular carcinoma	1 (13)

^a^Comorbidities without cancer diagnosis

^b^incl. 2 patients with medication for cancer treatment

Participating oncologists had a median of 10 years (range, 2–32) of professional experience. Ten out of 11 physicians (91%) found the issue of polypharmacy clinically relevant in terms of preventing side effects/complications and all physicians found reducing the number of medications and improving quality of life in cancer patients are important. 91% of physicians reported regularly using medication optimization tools such as Diagnosia^®^ or UpToDate^®^. The average time spent on medication history and review of polypharmacy at an initial presentation, was reported to be 10 (range, 3–15) and 5 (range, 1–10) minutes, respectively. About half of the participating physicians also reported querying non-prescription or alternative medications as part of the medication survey.

After the second part of the pilot test, physicians completed a questionnaire regarding the practicability of the TOP-PIC tool. All physicians rated TOP-PIC as helpful for patients affected by polypharmacy (0% not helpful, 40% rather helpful, 60% very helpful). In addition, oncologists were asked to estimate the time required versus the benefit to patients by using TOP-PIC. The time required was rated as rather high compared to the benefit for the patient (0% low, 30% moderate, 70% high). 64% of oncologists were positive about a regular use of TOP-PIC in everyday life.

The median time required to process a patient case was longer using TOP-PIC with 7 min (range, 2–28) compared to 5 min (range, 2–19) without the application of TOP-PIC (95% CI 1.7 to 3.5 min time difference; *P* < 0.001). In 81.8% of cases, the additional time spent on detailed medication review was less than 5 min. The time periods reported here for the application of TOP-PIC refer to steps 2 to 4, because retrospective patient data were processed in the pilot test (steps 1 and 5 require direct interaction with patients).

Across the testing, 56.3% of all drugs remained unchanged in the first and second part of the evaluation (without or with the use of TOP-PIC), 26.1% were changed equally in both parts, while 17.4% of medications had changes made in either the first or the second run. In the first part without using TOP-PIC, oncologists changed 33.3% of all medications in terms of discontinuing, reducing, increasing, replacing, or starting a drug. Most of the medication changes were discontinuations with 26.2%. When processing medications with TOP-PIC, significantly more medications were changed overall (36.5%; *P* = 0,016), and 29.9% of drugs were discontinued. For 17.4% of all medications, physicians made a different decision after using TOP-PIC than before. 8.6% of the drugs that were left unchanged during the first run were discontinued with the use of TOP-PIC. In contrast, 4.9% of the initially discontinued drugs were retained with the application of TOP-PIC. More detailed information about the drug changes can be found in Table S2 (Online Resource 2).

In terms of individual physicians, the number of medications changed varied widely in the first part (range 14–55 medications). In the second part, the behavior of individual physicians did not change significantly here (range 15–50 medications). Physicians with low change rates in the first run also made fewer changes in the second run. There was a strong positive correlation of the number of changed medications between the first and second part of the evaluation per physician (*r* = 0.83). Overall, these results reflect the individual decisions of different physicians, which can be changed only to a certain extent even by applying tools.

After classification of the drugs according to the Anatomical Therapeutic Chemical (ATC) classification system, it was evaluated in which groups changes were made most frequently. The most frequent changes in the first part could be observed in the groups of psychoanaleptics, antiphlogistics and antirheumatics, vitamins, antithrombotic agents and minerals (> 20 changed drugs absolutely per group). In the second part, in addition to the above groups, more than 20 changes were made in agents for acid-related diseases. With the use of TOP-PIC, an increase in drug changes of more than 10% was observed for the groups antianemics, vasoprotectors, antihistamines, lipid-lowering agents, and antiphlogistics and antirheumatics. The main substitutes were blood pressure medications (switching to combination preparations), analgesics (switching to potent opioids to replace tramadol or antiphlogistics and antirheumatics), and antithrombotic agents (switching from vitamin K antagonists to direct oral anticoagulants or heparin) in both parts.

An important question in the pilot test was whether physicians would be more confident in their decisions as a result of using TOP-PIC. In the first part, physicians were unsure in 9.3% of the decisions regarding medication changes, compared to only 4.8% in the second part (*P* = 0.001). If the decision was indicated as uncertain without the use of TOP-PIC, this was only the case in 23.7% after using TOP-PIC. In general, decision confidence was greater when a drug was maintained than when it was changed (93.3% in the first part, 98.0% in the second part, both for maintained drugs; *P* < 0.001). Uncertain decisions were significantly reduced with the use of TOP-PIC both for medications that were maintained and those that were changed (*P* < 0.001). Decision confidence increases significantly more in the group of changed medications compared to unchanged medications (23.9% vs 12.6%; *P* = 0.013). A summary of results of the pilot test is shown in Tables 4 and 5.

**Table 4:** Summary of outcomes of the pilot test without or with the use of TOP-PIC

	Without TOP-PIC	With TOP-PIC	Significance level
Median time required per patient case, min (range)	5 (2–19)	7 (2–28)	*P* < 0.001
Medication changes, *n* (%)	354 (33.3)	389 (36.5)	*P* = 0.016
Discontinued	279 (26.2)	318 (29.9)	
Dosage reduced	29 (2.7)	28 (2.6)	
Dosage increased	12 (1.1)	7 (0.7)	
Replaced	34 (3.2)	35 (3.3)	
Decisions classified as uncertain, *n* (%)	79 (9.3)	36 (4.8)	*P* = 0.001

**Table 5:** Certainty of treatment decisions in the pilot test

Decision confidence	Maintained medications	Changed medications	Significance level
High decision confidence after the use of TOP-PIC, *n* (%)	447 (98.0)	269 (90.9)	*P* < 0.001
Increase in decision certainty by type of decision, *n* (%)	71 (12.6)	33 (23.9)	*P* = 0.013

The second part of the pilot test also examined which step of the TOP-PIC tool was used for decisions regarding medication changes. Overall, TOP-PIC was used in 45.2% of all decisions. The second step was used most frequently in 33.8% (Screening for medication appropriateness), followed by the fourth step in 9.4% (TOP-PIC Disease-based list for benefit–risk assessment of medication) and the third step in 8.9% (Screening for drug interactions). When the TOP-PIC Disease-based list was used, it was considered helpful by oncologists in 94.5%.

## Discussion

Polypharmacy is a major problem not only in geriatric patients but also in patients with cancer. Comorbidities are frequently seen, and the number of medications increases with every additional chronic condition. As cancer therapy has changed in the last decades and patients even with incurable disease have a longer life expectancy, it is important to have a tool specifically designed to optimize pharmacotherapy in this patient group. Therefore, we developed the TOP-PIC tool consisting of five steps to optimize the medication of incurable cancer patients. The tool is designed for patients with a limited life expectancy of 3 years or less without an age limit. It incorporates medication appropriateness in general and considers the limited life span and time-to-treatment benefit. Furthermore, DDIs and risk for ADRs of long-term medication, immuno-/chemotherapeutic agents and concurrent medications are considered. The five-step procedure has a deliberate sequence to enable easy usage. At the same time, the step concept also allows TOP-PIC to be performed in a collaborative effort between clinical pharmacists and oncologists.

The pilot test conducted showed that by using TOP-PIC, different decisions were made for 17.4% of medications and that most medication changes were discontinuations. Thus, an overall reduction in medication can be assumed, which is certainly perceived as positive by the majority of patients. Another important finding from the pilot test is that the confidence in decision-making when changing medication was significantly improved by use of TOP-PIC. Therefore, the rate of evidence-based decisions may be increased by the TOP-PIC tool. Although a large proportion of the oncologists participating in the pilot test rated the time required as high compared with the benefit of TOP-PIC, 81.8% of the physicians invested less than 5 min of additional time, which is an acceptable time requirement in everyday clinical practice. In the pilot testing, the TOP-PIC Disease-based list was offered as a simple table without a search function. In clinical practice, a more user-friendly version of the tool will be offered including a search function, which should further reduce the time required for the user. In addition, with more frequent use of the TOP-PIC list, there will be a training effect so that the clinician will need to refer to it less frequently. The total time required to use TOP-PIC in the pilot test with retrospective patient cases refers to steps 2 to 4 of the tool. In the case of clinical application, the medical history taking and the final discussion with the patient must also be taken into account.

The TOP-PIC tool is innovative as it allows for a disease-based benefit–risk assessment of drugs and the usage of the tool directly by the treating oncologist. A benefit–risk assessment of taken drugs requires a specific approach for patients with an incurable disease. Some drugs for existing health conditions may no longer be clinically beneficial, because of a limited life expectancy. An innovation of our list is a precise listing of diseases and treatment recommendations for a patient group with a limited life span. In contrast, most other existing tools give advice for single drugs in terms of different lists of medications. In clinical practice, the utilization of a tool to optimize pharmacotherapy may be easier to handle if it is based on the patients’ diseases and not on single drugs, especially as patients often take several drugs for one disease. The included information and recommendations should facilitate decisions for clinicians, even beyond their specialty. The list not only offers assistance for adaptations of medications of all specialties, but also recommends consulting a specialist for particular diseases. It is important to note that every recommendation has to be seen in the context of the individual patient, the life expectancy, and the existing comorbidities. The treating physician has to decide on a case-by-case basis which drug can be stopped, maintained or started.

The two most similar existing approaches to optimizing medications of oncologic patients are the “OncPal deprescribing guideline” and the OncoSTRIP method (Lindsay et al. 2015; Vrijkorte et al. 2020). OncPal was designed for palliative patients with a very short life expectancy of 6 months. Unlike OncPal, our tool is applicable to a wider patient population as we include patients with a median life expectancy of up to 3 years. Furthermore, TOP-PIC provides information on the number needed to treat and the timespan until a benefit can be expected. With that information, the treating oncologist has the opportunity to estimate which medication changes are appropriate for the individual patient. Another tool, the OncoSTRIP, has been developed and published by a group from the Netherlands (Vrijkorte et al. 2020) based on a tool for geriatric patients (STRIP) (Drenth-van Maanen et al. 2018), which is embedded in the Dutch multidisciplinary polypharmacy guideline and is used in primary care. The method has been specified for older cancer patients ≥ 65 years with the aim to integrate it into routine care. This tool includes a precise polypharmacy anamnesis and pharmaceutical analysis, both performed by a pharmacist and a geriatric assessment performed by an oncology nurse. Interestingly, only 41% of the recommendations from the pharmacist were implemented by the treating physician after reviewing and discussing it with the patient (Vrijkorte et al. 2020). The suboptimal acceptance rate of recommendations seen in this study may be improved if the treating oncologist is directly involved in the medication review process, as with the TOP-PIC tool. A main difference between the OncoSTRIP method and our TOP-PIC tool is the structure of the benefit–risk assessment list, which is based on medications in the OncoSTRIP list and on indications or diseases in our list. Furthermore, our list is not limited to older patients and might, therefore, better suited to cancer patients with limited life expectancy of any age. A direct comparison of the described tools is not possible due to the different methodology. Nevertheless, the results appear to be comparable in terms of the proportion of drug changes. In the validation study of the OncPal desprescribing guideline, 123 of a total of 617 drugs (21.4%) were identified as PIMs (Lindsay et al. 2015). The use of OncoSTRIP reduced the total number of medications in 17 of 60 patients (28%) (Vrijkorte et al. 2020). In our pilot test, 389 of a total of 1065 medications were changed (36.5%) with application of TOP-PIC, and 318 of 1065 medications (29.9%) were discontinued. What the pilot test also shows is that even without an optimization tool, physicians make significant medication changes when explicitly asked to review a patient's history and medication list with regard to polypharmacy. Unfortunately, in clinical practice, too little attention is often paid to polypharmacy without the presence of a systematic approach. Another important aspect of the use of TOP-PIC demonstrated in the pilot test is a change in the decisions made based on evidence-based facts, as well as an increase in the confidence of physicians in making decisions.

An advantage of all deprescribing or medication-optimizing tools is a more detailed anamnesis of the patients’ medication and comorbidities and a more detailed physician–patient talk, which may result in improved compliance and adherence to the prescribed medication plan. Oncologic patients in particular appreciate taking part in every decision regarding their disease and medication. Therefore, the final shared decision-making process is of particular importance. The information from steps 2 to 4 of our tool assists the oncologist to come to an evidence-based decision, increases the certainty of decisions, and helps to explain the recommended medication changes to the patient.

A limitation of the TOP-PIC tool is that it has not been prospectively evaluated in a clinical trial. It might be difficult to prove efficacy and generate valid outcomes as a population of patients with incurable cancer receiving chemotherapy typically suffers from several health problems resulting from the malignant disease or the cancer treatment. Unplanned hospitalizations and deterioration of performance occur regularly as well as other complications like infections, thromboembolism, and bleeding events amongst others. Therefore, the usage of the TOP-PIC tool may be justified through clinical knowledge and an improvement of quality of life rather than traditional outcomes such as hospitalization rates or mortality. To address this, a prospective study is planned. The TOP-PIC Disease-based list will be reviewed and updated at regular intervals.

## Conclusion

Optimization of pharmacotherapy in patients with incurable cancer is an unmet need as the evidence for deprescribing potentially unnecessary drugs in cancer patients with a limited life expectancy is scarce. The TOP-PIC tool provides evidence-based facts and a five-step systematic approach to optimize medications and reduce the pill burden of incurable cancer patients. This assists treating oncologists in the everyday clinical decision-making process of drug prescription in oncologic patients with incurable cancer. Clinical effectiveness will be validated in a prospective clinical trial.

## Supplementary Information

Below is the link to the electronic supplementary material.

Supplementary file1 (PDF 419 KB)

Supplementary file2 (PDF 65 KB)

## Data Availability

The datasets used and analyzed during the current study are available from the corresponding author on reasonable request.

## Abbreviations

ADR: Adverse drug reaction
DDI: Drug–drug interaction
MAI: Medication appropriateness index
NNT: Number needed to treat
PIM: Potentially inappropriate medications
TOP-PIC: Tool to optimize pharmacotherapy in patients with incurable cancer
