# Supplementary material for: TOP-PIC: a new tool to optimize pharmacotherapy and reduce polypharmacy in patients with incurable cancer
Source: J Cancer Res Clin Oncol. 2023 Mar 6;149(10):7113–23. doi: 10.1007/s00432-023-04671-9 (PMC10374723; doi:10.1007/s00432-023-04671-9)
Supplement: Supplementary file 1 — Supplementary file1 (PDF 419 KB) [file 432_2023_4671_MOESM1_ESM.pdf]

Table S1 TOP-PIC Disease-based list for benefit-risk assessment of medications for cancer patients with limited life expectancy

Diseases (column 2) are categorized according to the specialty discipline (column 1, alphabetical order). Treatment recommendations can be found for the following disciplines: Cardiology, Dermatology, Electrolytes, Endocrinology, Gastroenterology, Gynecology, Hematology, Infectious Disease, Nephrology, Neurology, Ophthalmology, Orthopedics, Otolaryngology, Psychiatry, Pulmonology, Rheumatology, and Urology

**Specialty**	**Disease**	**Recommendation for cancer patients with limited life expectancy**	**Additional information/comment**	**ICD**	**Literature**
Cardiology	Acute coronary syndrome (ACS) within the last year (underlying coronary artery disease)	Dual antiplatelet therapy after ACS according to cardiological recommendation (PPI recommended)<br>Beta-blockers: Continuous therapy indicated after cardiogenic shock or additional cardiac or renal insufficiency.<br>Statin: 80 mg atorvastatin or 20 to 40 mg rosuvastatin<br>Antianginal therapy: see confirmed atherosclerotic cardiovascular disease (CVD)	Benefit of aspirin given for 5 years in preventing a new vascular event:<br>- Patients with ACS: NNT 20 in 6 months<br>- Patients after myocardial infarction, stroke or TIA: NNT 25 after 2-3 years<br><br>Benefits of statins given for 5 years for secondary prevention in patients with cardiovascular disease (CVD)<br>NNT 83 to save one life<br>NNT 39 to prevent one non-fatal heart attack<br>NNT 125 to prevent one stroke	I20.9, I21.9, I25.19, R07.4	UpToDate (19.12.2020)<br>- Coronary artery disease patients requiring combined anticoagulant and antiplatelet therapy<br>- Acute myocardial infarction: Role of beta blocker therapy<br>- Overview of the non-acute management of ST elevation myocardial infarction<br><br>http://www.thennt.com/ (13.02.2019)<br><br>PMID: 28886621<br>PMID: 26320110<br>PMID: 8054013
Cardiology	Aortic valve insufficiency	No established drug therapy; symptomatic therapy if necessary: preferred vasodilator: ACE inhibitor<br>Therapy of concomitant heart failure (see "Heart failure");	Unclear evidence of drug therapy with regard to remodeling<br><br>If surgical or interventional procedures are indicated due to the severity of the aortic valve stenosis, an interdisciplinary discussion should take place (cardiology, cardiac surgery and oncology)	I35.1	UpToDate (20.12.2020)<br>- Natural history and management of chronic aortic regurgitation in adults<br>- Vasodilator therapy in severe chronic aortic regurgitation in adults<br><br>PMID: 24603192<br>PMID: 28886619<br>PMID: 28315732
Cardiology	Aortic valve stenosis	No established drug therapy; symptomatic therapy if necessary<br><br>Cautious use of diuretics and beta-blockers; avoid vasodilators.<br>Good control of hypertension and heart failure; slow dosage titration to avoid hypotension.	If surgical or interventional procedures are indicated due to the severity of the aortic valve stenosis, an interdisciplinary discussion should take place (cardiology, cardiac surgery and oncology)	I35.0	UpToDate (19.12.2020)<br>- Medical management of symptomatic aortic stenosis<br>- Medical management of asymptomatic aortic stenosis in adults<br><br>PMID: 24603192<br>PMID: 28886619<br>PMID: 32201582<br>PMID: 28315732

Specialty	Disease	Recommendation for cancer patients with limited life expectancy	Additional information/comment	ICD	Literature
Cardiology	Arterial hypertension	Reduction/adaptation of antihypertensive drugs with the following target blood pressure:<br><160 mmHg systolic in primary prevention and low to moderate cardiovascular risk (≤ 2 risk factors).<br><140 mmHg systolic in manifest cardiovascular disease and/or high cardiovascular risk (≥ 3 risk factors).<br><br>Stricter blood pressure target in the presence of aortic aneurysm<br><br>ESC guideline risk factors: Male sex, age (men ≥55 years, women ≥65 years), dyslipidemia, elevated fasting glucose, obesity, positive family history of early cardiovascular disease.<br><br>A reduction of antihypertensives is often indicated in anemia.	Numerous anticancer drugs lead to an increase in hypertension, including cortisone, bevacizumab (risk >20%) and tyrosine kinase inhibitors.<br><br>Benefit in percent after 5 years (compared to no therapy):<br>97% no benefit<br>0.8% deaths prevented<br>1.5% strokes prevented<br>1.0% heart attacks/sudden cardiac deaths prevented<br><br>The benefit of blood pressure control is higher in patients with high baseline cardiovascular risk:<br>- Patients at very high cardiovascular risk: (5yr >21%): ARR 3.8% (NNT 26 for 5 years).<br>- Patients with low cardiovascular risk: (5yr ~6%): ARR 1.4% (NNT 71 for 5 years)<br><br>Patients with a target blood pressure <120 mmHg need on average one more antihypertensive drug than patients with a target blood pressure <140 mmHg.	I10.0	https://www.uptodate.com/ (19.12.2020)<br>- Overview of hypertension in adults<br>- Goal blood pressure in adults with hypertension<br>- Treatment of hypertension in older adults, particularly isolated systolic hypertension<br>- Can drug therapy be discontinued in well-controlled hypertension?<br><br>http://www.thennt.com/ (13.02.2019)<br><br>PMID: 25131978<br>PMID: 30234752<br>PMID: 24270181<br>PMID: 26551272<br>PMID: 31792659<br>PMID: 29475561<br>PMID: 30455322
Cardiology	Atrial fibrillation/flutter	Medical treatment identical to patients without cancer: Continue or initiate oral anticoagulation if CHA2DS2-VaSC score ≥2 (men) or ≥3 (women), as long as there is no high risk of bleeding. Direct oral anticoagulant (DOAC) preferred over Vitamin K antagonists (exceptions: mechanical heart valves).<br><br>Electrical cardioversion in symptomatic patients with a short history, LA < 5.5 cm and exclusion of atrial thrombus (TEE)<br><br>Regular electrolyte control, keep Hb >9 g/dl (ESA, EC)<br><br>Antiarrhythmics: purely symptomatic therapy; discontinuation of amiodarone/Multaq or other class I antiarrhythmic drugs in case of recurrence of atrial fibrillation after primary successful CV (prior level determination of amiodarone)<br><br>ß-blockers, verapamil-type Ca antagonists or digitalis for rate regulation:<br>- Symptomatic patient: target: resting pulse ≤80/min and pulse on (light) exertion ≤110 beats/min<br>- Asymptomatic patient: target: resting pulse ≤110/min	Benefit/harm of Warfarin in atrial fibrillation for 3 years:<br>NNT 13 Prevention of (primary) ischemic stroke<br>NNT 21 Prevention of death<br>NNH 13 Hemorrhage<br>NNH 192 intracerebral hemorrhage	I48.9	UpToDate (20.12.2020)<br>- Atrial fibrillation: Anticoagulant therapy to prevent thromboembolism<br>- Rhythm control versus rate control in atrial fibrillation<br>- Control of ventricular rate in atrial fibrillation: Pharmacologic therapy<br>- Atrial fibrillation: Risk of embolization<br><br>http://www.thennt.com/ (13.02.2019)<br><br>PMID: 27567408<br>PMID: 24685669<br>PMID: 16034869<br>PMID: 30686041<br>PMID: 32860505

Specialty	Disease	Recommendation for cancer patients with limited life expectancy	Additional information/comment	ICD	Literature
Cardiology	Confirmed atherosclerotic cardiovascular disease (CVD) including coronary artery disease (CAD), cerebrovascular disease (CVD) and peripheral artery disease (PAD)	Antiplatelet therapy: aspirin 100mg OD or clopidogrel 75mg OD<br>PPI in the presence of the following risk factors: post GI-ulcer/GI-bleeding, concurrent anticoagulant, NSAID or corticosteroid therapy; more than two of the following risk factors: ≥65 years, dyspepsia, gastroesophageal reflux disease, known Helicobacter pylori-infection or chronic alcohol abuse.<br><br>Statin therapy is indicated.<br><br>CAD: Discontinuation of antianginal therapy is possible in asymptomatic patients (no influence on mortality).<br>Antianginal therapy: 1st choice: short-acting nitrate + beta-blocker or calcium antagonist (if both are not possible: dihydropyridine-type calcium antagonist (e.g. amlodipine); in AP CCS > 2: combine beta-blocker + amlodipine);<br>2nd choice (additional or change to): Ivabradine (precondition: sinus rhythm), long-acting nitrate (observe nitrate-free interval), Nicorandil, Ranolazine, Trimetazidine.<br><br>PAD: Attempt to discontinue symptomatic claudication therapy in the absence of clinical improvement.	Beta-blockers, calcium antagonists and nitrates are only symptomatic.<br><br>Aspirin vs placebo<br>Vascular event: 6.7% vs 8.2% per year (NNT 66/year)<br>Stroke 2.1% vs 2.5% per year (NNT 217/year)<br>Coronary events 4.3% vs 5.3% per year (NNT 100/year)<br><br>Aspirin for 5 years in patients with CVD<br>NNT 50 in preventing one additional cardiovascular problem<br>NNT 333 in preventing one additional death<br>NNH 400 Severe bleeding<br><br>Statins for 5 years in patients with CVD<br>NNT 83 in preventing an additional death<br>NNT 39 in preventing non-fatal myocardial infarction<br>NNT 125 in preventing stroke<br><br>PAD<br>Naftidrofuryl: improvement in pain-free walking distance: NNT 4.5<br>Improvement in pain-free walking distance compared with placebo:<br>Naftidrofuryl 49%, Cilostazol 13%, Pentoxifylline 9%	I20.9, I21.9, I25.19, R07.4	UpToDate (19.12.2020)<br>- Overview of the prevention of cardiovascular disease events in those with established disease (secondary prevention) or at very high risk<br>- Aspirin for the secondary prevention of atherosclerotic cardiovascular disease<br>- Antiplatelet therapy for the secondary prevention of ischemic stroke<br>- Overview of secondary prevention of ischemic stroke<br>- Overview of lower extremity peripheral artery disease<br>- Management of claudication due to peripheral artery disease<br><br>http://www.thennt.com/ (13.02.2019)<br><br>PMID: 23996286<br>PMID: 23166210<br>PMID: 19482214<br>PMID: 23032550<br>PMID: 23235580<br>PMID: 23034699<br>PMID: 27851992

Specialty	Disease	Recommendation for cancer patients with limited life expectancy	Additional information/comment	ICD	Literature
Cardiology	Heart failure	Primary prevention: Cardiac monitoring in cancer drugs with an increased risk of heart failure, e.g. anthracyclines and antibodies against Her2neu; prophylactic heart failure therapy is not recommended.<br><br>Preexisting or new onset heart failure during tumor therapy EF ≥40%: Fluid restriction <2l l/day, weight measurement, diuretic therapy (primarily loop diuretics) in minimally required doses as symptomatic therapy (dyspnea, oedema); no proven efficacy (= reduction of mortality) through neurohumoral therapy but in case of comorbidity e.g. hypertension or diabetes, ACE inhibitors/ARB are to be preferred; i.v. iron administration and compensation of anemia improves symptoms and reduces hospitalization.<br><br>EF <35-40%: therapy as for EF ≥ 40%, in addition ACE inhibitors and ß-blockers; in case of persistent symptoms additional mineralocorticoid receptor antagonists (MRA, e.g. spironolactone 25 mg), taking renal function into account.<br><br>If the course is stable (max. NYHA I-II), try discontinuing MRA for several months. In case of diuretic therapy and hypokalemia, consider combination drug.<br><br>If heart failure symptoms persist (≥NYHA II) consultation with the cardiologist in charge: exchange of ACE inhibitor/ARB for therapy with sacubitril/valsartan (Entresto®) or additionally Ivabradine (prerequisite: Sinus rhythm + heart rate >70/min)<br><br>Cardiac resynchronization therapy (CRT device) should be discussed in individual cases with a corresponding life expectancy if symptoms persist despite optimal drug therapy. Prerequisite: LVEF ≤35 % and QRS duration ≥130 ms and left bundle branch block.	Not recommended, among others: Glitazones, NSAID/COX2 inhibitors, diltiazem, verapamil, antiarrhythmics (except amiodarone).<br><br>Risk/Benefit Ratio<br>ACE inhibitors: patients with heart failure and LVEF ≤ 35%; endpoint: mortality after 4 years: Enalapril 35.2%, placebo 39.7%, NNT 22<br><br>Heart failure grade III/IV on therapy with ACE inhibitors, diuretics; randomization: additionally, spironolactone or placebo. Results after 2 years:<br>- Reduction in mortality (35% deaths on spironolactone vs 46% on placebo; NNT 10) and risk of cardiovascular-related hospital admission (32% on spironolactone vs 40% on placebo; NNT 12).<br>- Side effects: Gynecomastia or chest pain (10% for spironolactone vs 1% for placebo; NNH 11).<br>- No difference in risk of severe hyperkaliemia (potassium ≥6 mmol/L).	I25.5, I34.0, I42.7, I50.00, I50.01, I50.11, I50.12, I50.13, I50.14, I50.9	UpToDate (19.12.2020)<br>- Overview of the management of heart failure with reduced ejection fraction in adults<br>- Initial pharmacologic therapy of heart failure with reduced ejection fraction in adults<br>- Treatment and prognosis of heart failure with preserved ejection fraction<br>- Cardiotoxicity of non-anthracycline cancer chemotherapy agents<br><br>PMID: 27206819<br>PMID: 23741058<br>PMID: 1414890<br>PMID: 10471456
Cardiology	Hypercholesterolemia	See chapter "Primary prevention of a cardiovascular event in the case of unconfirmed vascular disease"		E78.0	
Cardiology	Hypertriglyceridemia	Continue fibrate therapy in case of severe HTG, otherwise discontinue.<br>In case of known severe hypertriglyceridemia or after acute pancreatitis, monitor triglyceride levels after discontinuation of medication.<br><br>In case of borderline triglyceride levels (related to the risk of pancreatitis) and prolonged cortisone therapy, monitoring of triglyceride levels is advisable.	Risk of acute pancreatitis approx. 5% with triglycerides >1000 mg/dL and 10-20% with triglycerides >2000 mg/dL<br>The risk of pancreatitis is low with non-severe HTG.<br><br>The cardiovascular risk is better reduced with statins.	E78.1	UpToDate (18.12.2020)<br>- Hypertriglyceridemia<br>- Hypertriglyceridemia-induced acute pancreatitis<br><br>PMID: 22962670<br>PMID: 24731657<br>PMID: 31504418<br>PMID: 27849333<br>PMID: 26497361<br>PMID: 30083325

Specialty	Disease	Recommendation for cancer patients with limited life expectancy	Additional information/comment	ICD	Literature
Cardiology	Mitral valve insufficiency	No prophylactic drug therapy; symptomatic therapy, if necessary<br>Therapy of concomitant heart failure (see "Heart failure")	If, due to the severity of the mitral valve insufficiency, a surgical or interventional procedure is indicated, an interdisciplinary discussion should take place (cardiology, cardiac surgery and oncology).	I34.0	UpToDate (19.12.2020)<br>- Management of chronic primary mitral regurgitation<br><br>PMID: 24603192<br>PMID: 28886619<br>PMID: 28315732<br>PMID: 29447328
Cardiology	Mitral valve stenosis	Drug therapy only in case of symptomatic heart failure (diuretics, beta-blockers; see "Heart failure").<br>Contraindication to ACE inhibitors/AT-II blockers<br>Anticoagulation in case of atrial fibrillation, post embolic event or left ventricular thrombus	If surgery or interventional procedures are indicated due to the severity of the mitral regurgitation, an interdisciplinary discussion should take place (cardiology, cardiac surgery and oncology).<br><br>Follow-up by transthoracic echocardiography depending on severity: every 3-5 years for valve orifice area > 1.5 cm², every 1-2 years for valve orifice area 1.0-1.5 cm², 1x/year for valve orifice area < 1.0 cm².	I34.2	UpToDate (20.12.2020)<br>- Overview of the management of mitral stenosis<br><br>PMID: 24603192<br>PMID: 28886619<br>PMID: 28315732
Cardiology	Primary prevention of cardiovascular event in high-risk patients without cardiovascular disease including coronary artery disease (CAD), cerebrovascular disease (CVD) and peripheral artery disease (PAD)	Stop aspirin (no indication)<br>Discontinue statin in low and moderate cardiovascular risk or life expectancy <1 year or in renal replacement therapy.<br><br>Continue statin if at high risk and life expectancy > 1 year:<br>- Diabetes mellitus with end-organ damage (e.g. proteinuria) or severe risk factors such as nicotine abusus, severe hyperlipidemia or severe hypertension<br>- Chronic renal failure ≥ stage G3a (GFR <60 mL/min)<br>- Cholesterol >310 mg/dL<br>- Hypertension III°<br>- ESC risk score > 5% above 10a	Assess cardiovascular risk according to ESC Guidelines/SCORE<br><br>Aspirin vs placebo:<br>Vascular event: 0.51% vs 0.57% per year (NNT 1667/year).<br>Stroke: no benefit<br>Death from vascular event: no benefit<br>NNH on major bleeding: 3333<br><br>Statins vs placebo for 5 years<br>- No death prevented<br>- Heart attack NNT 60<br>- Stroke NNT 268<br>- Development of diabetes NNH 50		UpToDate (28.12.2020)<br>- Overview of primary prevention of cardiovascular disease<br>- Cardiovascular disease risk assessment for primary prevention: Our approach<br>- Aspirin in the primary prevention of cardiovascular disease and cancer<br>- Cardiovascular disease risk assessment for primary prevention: Risk calculators<br><br>http://www.thennt.com/ (13.02.19)<br><br>PMID: 27222591<br>PMID: 19482214<br>PMID: 25798575<br>PMID: 31504418<br>PMID: 31365087<br>PMID: 30879355<br>PMID: 31497854
Cardiology	Pulmonary hypertension	Review indication, effectiveness, potential interactions/side-effects with planned tumor therapy to specific PAH therapy.<br>Basic therapy: diuretics, anticoagulation for chronic thromboembolic pulmonary hypertension, verapamil for supraventricular tachycardia.<br>Close clinical monitoring and change of current therapy only after consultation with the treating specialist.	Benefit of anticoagulation in primary pulmonary hypertension (PAH group 1) is controversial.<br><br>Significant reduction in mortality under drug therapy: 1.5% mortality under PAH therapy vs. 3.8% mortality under placebo (with an average observation period of only 14.3 weeks (NNT 62)	I27	UpToDate (19.12.2020)<br>- Treatment of pulmonary arterial hypertension (group 1) in adults: Pulmonary hypertension-specific therapy<br>- Pulmonary hypertension due to lung disease and/or hypoxemia (group 3 pulmonary hypertension): Treatment and prognosis<br>- Overview of the treatment of chronic thromboembolic pulmonary hypertension<br>- Clinical features and diagnosis of pulmonary hypertension of unclear etiology in adults<br><br>PMID: 26320113<br>PMID: 19155250<br>PMID: 19389575<br>PMID: 30545968

Specialty	Disease	Recommendation for cancer patients with limited life expectancy	Additional information/comment	ICD	Literature
Cardiology	Sinus bradycardia/Sick Sinus Syndrome (SSS)	Balance electrolytes, exclude hypothyroidism<br>Asymptomatic including asymptomatic SSS: no therapy recommended<br>Symptomatic SSS: discontinue bradycardia-inducing drugs if justifiable, otherwise pacemaker implantation<br>In case of persistence of symptomatic SSS: pacemaker implantation if life expectancy >1 year		R00.1, I49.5	UpToDate (25.12.2020)<br>- Sinus bradycardia<br>- Sinus node dysfunction: Treatment<br><br>PMID: 30412709<br>PMID: 9236443<br>PMID: 23801827
Cardiology	Sinus tachycardia, atrial tachycardia, AVNRT without pre-excitation syndrome, palpitations	Beta blocker/calcium channel blocker for symptoms<br>Ablation treatment for persistence or ineffectiveness of medication		I47.1, I47.2, R00.0, R00.2	UpToDate (20.12.2020)<br>- Sinus tachycardia: Evaluation and management<br>- Overview of the acute management of tachyarrhythmias<br>- Evaluation of palpitations in adults<br><br>PMID: 26399663<br>PMID: 27856540<br>PMID: 21697315<br>PMID: 31837143
Cardiology	Symptomatic hypotension	Discontinue medications that promote hypotension.<br>Correct anemia (see Anemia), exclude adrenocortical insufficiency, ensure adequate fluid intake<br>Fludrocortisone (Astonin H) for orthostatic hypotension; if symptom control is inadequate, an alpha-1 agonist (e.g. midodrine) can be added; if there is no improvement, discontinue therapy.<br>Caffeine, pyridostigmine (mestinone) may be used as adjunct therapy.	Little evidence of a significant effect of etilefrin hydrochloride.	I95.9	UpToDate (20.12.2020)<br>- Mechanisms, causes, and evaluation of orthostatic hypotension<br>- Treatment of orthostatic and postprandial hypotension<br><br>PMID: 25150287<br>PMID: 23472781<br>PMID: 23458585<br>PMID: 22591985<br>PMID: 27372462<br>PMID: 29562304
Cardiology	Venous thromboembolism	If long-term anticoagulation due to VTE was already indicated before the cancer diagnosis (spontaneous proximal venous thrombosis or spontaneous pulmonary embolism), therapeutic anticoagulation should be continued unless there is a contraindication. Patients being anticoagulated with a vitamin K antagonist should be switched to a DOAC in case that time in therapeutic range is below 75% (INR).<br>In case of secondary venous thromboembolism (surgery, trauma,...) more than 3 months ago, anticoagulation should be discontinued.<br><br>In the case of thrombosis within 3 months before or after cancer diagnosis, a causal association with the tumor disease must be assumed (cancer-associated thrombosis): Anticoagulation should be performed as long as there is no contraindication (preferably low molecular weight heparin, edoxaban or rivaroxaban).<br><br>Primary prophylaxis: Cancer and drug treatment of tumors increase the risk of venous thromboembolism. If the risk of thrombosis is high (Khorana Score >3), primary thrombosis prophylaxis can be considered.	Warfarin vs. placebo: The risk of recurrent thrombosis/embolism after a first spontaneous venous thromboembolism (proximal venous thrombosis and/or pulmonary embolism) 2 years after discontinuation of anticoagulation is 15% compared to 1.3% with continued anticoagulation (NNT 8).	I80.9, I87.00	UpToDate (20.12.2020)<br>- Venous thromboembolism: Anticoagulation after initial management<br>- Overview of the treatment of lower extremity deep vein thrombosis (DVT)<br>- Rationale and indications for indefinite anticoagulation in patients with venous thromboembolism<br>- Anticoagulation therapy for venous thromboembolism (lower extremity venous thrombosis and pulmonary embolism) in adult patients with malignancy<br><br>PMID: 31492632<br>PMID: 31381464<br>PMID: 10089183<br>PMID: 12853587<br>PMID: 29920657<br>PMID: 31697840<br>PMID: 29231094<br>PMID: 29746227

Specialty	Disease	Recommendation for cancer patients with limited life expectancy	Additional information/comment	ICD	Literature
Cardiology	Ventricular tachycardia	No change in ongoing therapy with good control; check electrolytes regularly.<br>Avoid substances with cardiotoxic risk such as prolongation of the QT interval<br><br>For premature ventricular extrasystole and non-sustained VT (<10 beats), there is no indication for continuous antiarrhythmic therapy as long as the patient is asymptomatic and if there is no heart failure or post-myocardial infarction.<br><br>For ICD: amiodarone + beta-blocker best to prevent shocks<br>In symptomatic patients, discuss ablation treatment.	Survived ventricular fibrillation or death from arrhythmia: amiodarone versus placebo: Relative risk reduction 48.5%, NNT 37	I47.2	UpToDate (20.12.2020)<br>- Nonsustained ventricular tachycardia: Clinical manifestations, evaluation, and management<br>- Sustained monomorphic ventricular tachycardia in patients with structural heart disease: Treatment and prognosis<br><br>PMID: 25172618<br>PMID: 26320108<br>PMID: 29084731<br>PMID: 9078198<br>PMID: 7539890<br>PMID: 16403928
Dermatology	Chronic venous insufficiency, varicosities, postthrombotic syndrome	Discontinuation of all venotonics and rheologics<br>Discontinue diuretics if no other indication<br>Continue or initiate compression stocking therapy in case of clinical benefit	Phlebotonics may have positive effects on oedema, trophic disturbances, cramps and paresthesia, but there are more side effects compared to placebo. Phlebotonics do not show a higher rate of ulcer healing compared to placebo.<br><br>Risk reduction for oedema RR 0.7, ulceration RR 0.94	I87.20, I83.9, I87.00	UpToDate (20.12.2020)<br>- Medical management of lower extremity chronic venous disease<br>- Compression therapy for the treatment of chronic venous insufficiency<br>- Post-thrombotic (postphlebitic) syndrome<br><br>S3-Guideline on Venous Leg Ulcer<br>Developed by the Guideline Subcommittee ‘Diagnostics and<br>Treatment of Venous Leg Ulcers’ of the European Dermatology Forum<br><br>PMID: 28211296<br>PMID: 25246013<br>PMID: 24323411<br>PMID: 28549402<br>PMID: 27048768<br>PMID: 8569363<br>PMID: 29385792
Dermatology	Pruritus	Local therapy according to dermatological recommendation (e.g. topical steroids, capsaicin, topical calcineurin inhibitors, topical anesthetics).<br>Adequate skin care to avoid dry skin<br>If systemic therapy is necessary 1st choice antihistamines<br>If pruritus is severe, treatment with antidepressants or gabapentin or pregabalin may be tried<br>Cholestyramine for icteric pruritus	Opioid receptor antagonists can be effective, but often have side effects and are contraindicated in ongoing opioid-containing pain therapy.<br><br>If opioid-induced pruritus is suspected, try switching to another opioid preparation.	L29.9	UpToDate (28.12.2020)<br>- Pruritus: Overview of management<br>- Pruritus associated with cholestasis<br>- Overview of pruritus and sweating in palliative care<br>- Uremic pruritus<br><br>European Guideline on Chronic Pruritus<br>In cooperation with the European Dermatology Forum (EDF) and the European Academy of Dermatology and Venereology (EADV), 2019<br><br>PMID: 28466423<br>PMID: 30530232

Specialty	Disease	Recommendation for cancer patients with limited life expectancy	Additional information/comment	ICD	Literature
Dermatology	Psoriasis	Regular evaluation of the Psoriasis Area and Severity Index score (PASI 90).<br>Maintain or, if necessary, optimize local therapy;<br>Discontinuation of a beta-blocker, if justifiable.<br>Improvement of symptoms is possible through immunosuppressive tumor therapy/adjuvant therapy, so that immunomodulatory systemic therapies can possibly be reduced.	Attention should be paid to a possible interaction resulting in increased side effects (e.g. diarrhea or nausea with apremilast).	L40.9	UpToDate (25.12.2020)<br>- Epidemiology, clinical manifestations, and diagnosis of psoriasis<br>- Treatment of psoriasis in adults<br>- Treatment of psoriatic arthritis<br><br>PMID: 26749174<br>PMID: 21306785<br>PMID: 26644232<br>PMID: 26481193<br>PMID: 24990147
Dermatology	Urticaria	Regular evaluation of the symptoms, also with regard to allergic reactions (asthma,...). The tumor disease or tumor therapy (e.g. AK therapy) or concomitant therapy can improve or aggravate the symptoms.<br>Avoid NSAIDs.<br>H1 antihistamines of the 2nd generation to be preferred; H2 blockers if symptom control is insufficient (discontinue after 4 weeks if ineffective).<br>Local therapy with steroids possible<br>Omalizumab in severe chronic urticaria; if there is a response, try tapering after one year at the earliest.	In urticaria, up to 4 times higher dosage of H1 antihistamines is justified (e.g. levocetiricin 20mg/d).<br><br>In case of aggravation with initiation of tumor therapy, evaluate alternative tumor therapy.	L50.9	UpToDate (25.12.2020)<br>- New-onset urticaria<br>- Chronic spontaneous urticaria: Clinical manifestations, diagnosis, pathogenesis, and natural history<br>- Chronic spontaneous urticaria: Standard management and patient education<br>- Chronic spontaneous urticaria: Treatment of refractory symptoms<br><br>PMID: 29336054<br>PMID: 24766875<br>PMID: 25397904<br>PMID: 22419335<br>PMID: 27237730
Electrolytes	Chronic hyperkalemia	Potassium values > 6.5 mmol/l or symptomatic moderate hyperkalemia is an emergency and is treated according to the guidelines.<br><br>In case of potassium values >5.5 mmol/l, check the triggering medication and discontinue if possible.<br>Loop diuretics or thiazide diuretics should be used in patients with hypervolemia.<br>Metabolic acidosis and insulin deficiency (poorly controlled diabetes) should be compensated.<br>Partiromer (2. choice: Sodium Polystyrene Sulfonate) at potassium values >5.5 mmol/l	In stable and asymptomatic patients, higher potassium values can be tolerated in individual cases.<br><br>Sodium Polystyrene Sulfonate has a more adverse medication profile than Partiromer and is not recommended for long-term therapy.	E87.5	UpToDate (18.12.2020)<br>- Treatment and prevention of hyperkalemia in adults<br>- Causes and evaluation of hyperkalemia in adults<br>- Clinical manifestations of hyperkalemia in adults<br><br>PMID: 29244647<br>PMID: 26371733<br>PMID: 27600582<br>PMID: 28778861<br>PMID: 33160639
Electrolytes	Hypercalcemia	Discontinuation of calcium and vitamin D preparations<br>No thiazides<br>Observation without therapy in mild hypercalcemia/asymptomatic patients (calcium <3 mmol/l)<br>Drug therapy in cases of severe hypercalcemia and symptoms: preferably i.v. bisphosphonate, in case of contraindication denosumab (acute: calcitonin)<br><br>See also "Primary hyperparathyroidism"	Regular calcium monitoring after bisphosphonate or denosumab administration, as hypocalcemia may occur after therapy<br><br>Add i.v. fluid (NaCl 0.9%) to calcium lowering therapy, loop diuretics only in case of hypervolemia after fluid administration	E83.59	UpToDate (28.12.2020)<br>- Treatment of hypercalcemia<br>- Hypercalcemia of malignancy: Mechanisms<br><br>PMID: 27170690<br>PMID: 27588937<br>PMID: 31826272

Specialty	Disease	Recommendation for cancer patients with limited life expectancy	Additional information/comment	ICD	Literature
Electrolytes	Hypokalemia	Potassium values < 3.0 mmol/l or symptomatic hypokalemia is an emergency and is treated according to the guidelines.<br><br>Treatment of mild to moderate hypokalemia (3.0-3.4 mmol/l) depends upon the cause of the hypokalemia and acid-base status:<br>Check the triggering medication and discontinue if possible;<br>Potassium-rich diet<br>In case of diarrhea/vomiting: potassium chloride (especially in metabolic alkalosis) or potassium bicarbonate (2nd choice); temporary intravenous potassium substitution if oral intake is not possible.<br>In renal potassium wasting (e.g. chronic diuretic therapy, renal tubular acidosis, hypomagnesemia, abiraterone) a potassium-sparing diuretic such as amiloride is likely to be more effective than potassium substitution.<br>In case of primary aldosteronism: spironolactone	Determining whether hypomagnesemia is present is important because hypokalemia often cannot be corrected until the magnesium deficit is reversed.<br><br>Pay attention to the amount of potassium in the different preparations.	E87.6	UpToDate (18.12.2020)<br>- Clinical manifestations and treatment of hypokalemia in adults<br>- Evaluation of the adult patient with hypokalemia<br>- Causes of hypokalemia in adults<br><br>PMID: 26934393<br>PMID: 9700180<br>PMID: 10979053<br>PMID: 12401935<br>PMID: 29540487
Endocrinology	Diabetes mellitus type-1, New onset insulin-dependent Diabetes type-3c (pancreatogenic diabetes)	Therapy goal: HbA1c <8 mg% (strive for stricter goal in case of secondary complications).<br>Keep therapy regime if the patient is well adjusted<br>Close monitoring of blood glucose levels during chemotherapy/cortisone	Before changes in therapy always take into account the risk of hypoglycemia as well as the severity and frequency of previous blood glucose imbalances.<br><br>Higher HbA1c values may be accepted after individual consideration.	E10.0-E10.9	UpToDate (18.12.2020)<br>- Overview of general medical care in nonpregnant adults with diabetes mellitus<br>- Management of blood glucose in adults with type 1 diabetes mellitus<br>- Glycemic control and vascular complications in type 1 diabetes mellitus<br><br>PMID: 31497854<br>PMID: 25114297<br>PMID: 24526393<br>PMID: 28594069<br>PMID: 29222377
Endocrinology	Diabetes mellitus type-2	Therapy goal: HbA1c < 8 mg% (strive for stricter goal in case of manifest secondary diseases), avoidance of hypoglycemia<br>Keep therapy regime if the patient is well adjusted<br>Close monitoring of blood glucose levels during chemotherapy/cortisone<br>Oral therapy only: attempt to reduce dosage<br>Combined therapy with insulin plus oral agents: aim for the simplest possible therapy regime	Higher HbA1c values may be accepted after individual consideration.	E11.0-E11.9	UpToDate (18.12.2020)<br>- Initial management of blood glucose in adults with type 2 diabetes mellitus<br>- Glycemic control and vascular complications in type 2 diabetes mellitus<br>- Treatment of type 2 diabetes mellitus in the older patient<br><br>PMID: 24731476<br>PMID: 29507945<br>PMID: 31497854<br>PMID: 28594069<br>PMID: 29222377<br>PMID: 30110583<br>PMID: 20594588<br>PMID: 24214280

Specialty	Disease	Recommendation for cancer patients with limited life expectancy	Additional information/comment	ICD	Literature
Endocrinology	Hyperthyroidism	TSH and fT3/fT4 control intervals according to the recommendation of the special outpatient clinic.<br>Continuation of ongoing thyreostatic therapy in the case of previously known manifest hyperthyroidism. Regular follow-up including TSH, fT3/fT4, blood count, liver function parameters.<br>Newly diagnosed hyperthyroidism: complete assessment of thyroid function in a special outpatient clinic and evaluation of medical treatment options (thionamides, radioiodine); treatment should be started in manifest hyperthyroidism with de novo synthesis of hormone (Hyperthyreoidism with normal or high radioiodine uptake), but not in decay hyperthyroidism).<br><br>Discontinue beta blockers if there are no symptoms and no indication other than hyperthyroidism.	Elderly patients often have no classic symptoms, but are rather apathetic.<br><br>Low TSH and low fT3/fT4 is seen with high-dose cortisone therapy<br><br>Sodium perchlorate before iodine-containing contrast agents administration; watch out for side effects such as skin rash, arthralgia, hepatotoxicity, bone marrow suppression	E05.9	UpToDate (18.12.2020)<br>- Diagnosis of hyperthyroidism<br>- Subclinical hyperthyroidism in nonpregnant adults<br>- Disorders that cause hyperthyroidism<br>- Beta blockers in the treatment of hyperthyroidism<br><br>PMID: 27521067<br>PMID: 30283735<br>PMID: 26558232<br>PMID: 29081800<br>PMID: 22529182<br>PMID: 20091544
Endocrinology	Hyperuricemia, gout	Continue medication if genetic disease is known.<br>Attempt to discontinue uric acid-lowering therapy in the absence of organ damage and with little or no history of symptoms (rare and mild gout attacks, <2 attacks/ year).<br>Initiation or continuation of uric acid-lowering therapy in the case of uricemia-related organ damage (urate kidney stones, radiologically detectable uric acid crystals, GFR < 60 ml/min, tophi, joint damage) or in the case of frequent gout attacks (> 1 attack/year)<br>Uric acid target value: <6mg/dl or <5mg/dl in tophus/polyarticular gout.<br>First choice: allopurinol; start with low dose, slowly increase dose until target value is reached.<br><br>Target uric acid levels under cytostatic therapy:<br>< 6mg/dl in nephrotoxic tumor therapy<br>< 7mg/dl for non-nephrotoxic tumor therapy<br>Allopurinol in the initial treatment phase in case of increased tumor lysis risk (possibly also rasburicase)	Febuxostat is more potent in lowering uric acid, but increased rate of cardiovascular events<br>Dose reduction of allopurinol in renal insufficiency (GFR <60 ml/min)	E79.0, M10	UpToDate (18.12.2020)<br>- Lifestyle modification and other strategies to reduce the risk of gout flares and progression of gout<br>- Treatment of gout flares<br>- Pharmacologic urate-lowering therapy and treatment of tophi in patients with gout<br>- Asymptomatic hyperuricemia<br><br>PMID: 23868909<br>PMID: 27802508<br>PMID: 23024028<br>PMID: 23024029<br>PMID: 27457514<br>PMID: 25314636<br>PMID: 32390306
Endocrinology	Hypothyroidism	TSH and fT3/fT4 at 3-6 months intervals or when clinical symptoms occur.<br>Maintain current therapy, therapy goal: TSH in normal range.<br>Newly diagnosed hypothyroidism: absolute indication for therapy with TSH >10 mIE/L or manifest hypothyroidism (low fT4).<br>With TSH 4-10 mIE/L and symptoms: therapy attempt (but if there is no improvement despite TSH normalization, therapy should be discontinued).	Recommendations refer to primary hypothyroidism; secondary or central hypothyroidism should be excluded; special features in cancer patients: possible cerebral metastasis with disturbance of the central control circuit, ongoing immunotherapy with possibility of central and peripheral disturbances (hypophysitis, thyroiditis); central disturbances often show failures of several hormones/control circuits.<br><br>Especially in older patients, a TSH in the upper normal range up to low latent hypothyroidism is tolerable.<br><br>If weight loss exceeds 10%, dose reduction is often necessary	E03.9	UpToDate (18.12.2020)<br>- Treatment of primary hypothyroidism in adults<br>- Clinical manifestations of hypothyroidism<br>- Subclinical hypothyroidism in nonpregnant adults<br>- Toxicities associated with checkpoint inhibitor immunotherapy<br>- Central hypothyroidism<br><br>PMID: 31088853<br>PMID: 24783053<br>PMID: 26010808<br>PMID: 25266247<br>PMID: 20858880<br>PMID: 33064663<br>PMID: 28402245

Specialty	Disease	Recommendation for cancer patients with limited life expectancy	Additional information/comment	ICD	Literature
Endocrinology	Osteoporosis	Maintain or start medication in cases of manifest osteoporosis, post osteoporotic hip or vertebral fracture or osteopenia with a high risk of fracture (bisphosphonate/denosumab, calcium, vitamin D).<br>No medication in osteopenia without additional risk factors such as endocrine therapy.<br>In case of short life expectancy of a few months, discuss discontinuation of osteoporosis therapy with the patient.<br>In case of chronic kidney failure stage G4 (GFR < 30 ml/min), osteoporosis therapy only at the recommendation of a nephrologist.	WHO-definition of high fracture risk: ≥3% 10-year probability of hip fracture or ≥20% of other osteoporotic fracture (Fracture Risk Assessment Algorithm, FRAX).<br><br>Primary prevention of hip fractures in older, high-risk adults with calcium and vitamin D: NNT 111/year<br>Secondary prevention of new fractures during therapy with zoledronic acid: NNT 20 /year	M80.99, M81.99	UpToDate (26.12.2020)<br>- Overview of the management of osteoporosis in postmenopausal women<br>- Treatment of osteoporosis in men<br>- Denosumab for osteoporosis<br>- Calcium and vitamin D supplementation in osteoporosis<br>- The use of bisphosphonates in postmenopausal women with osteoporosis<br><br>DVO-LEITLINIE 2017 zur Prophylaxe, Diagnostik und Therapie der Osteoporose bei postmenopausalen Frauen und bei Männern<br><br>PMID: 30907953<br>PMID: 22675062<br>PMID: 28492856<br>PMID: 25182228<br>PMID: 24729336<br>PMID: 17878149<br>PMID: 30568435<br>PMID: 28789921
Endocrinology	Primary hyperparathyroidism	Known primary HPT: Continue current therapy<br>In the case of bone metastases, switch from cinacalcet to bisphosphonate without calcium substitution.<br><br>Initial diagnosis: therapy with cinacalcet in cases of severe hypercalcemia and/or symptoms, if surgery is not possible or advisable; if osteoporosis or bone metastases are also present, therapy with bisphosphonate.<br>Vitamin D substitution (400 - 800 IU/d) if 25-hydroxyvitamin D [25(OH)D] ≤20 ng/mL.<br>In asymptomatic patients, an observation phase without therapy is also justifiable (for approx. 1 year).	In the case of very high calcium levels, bisphosphonate therapy can be useful even without osteoporosis or bone metastases.<br><br>In case of renal insufficiency, denosumab may be considered as an alternative.<br><br>In addition to calcium concentration, other symptoms/complications should also be evaluated, such as kidney stones, nephrocalcinosis, renal insufficiency, neurological symptoms.	E21.0	UpToDate (18.12.2020)<br>- Primary hyperparathyroidism: Diagnosis, differential diagnosis, and evaluation<br>- Primary hyperparathyroidism: Management<br><br>PMID: 25162665<br>PMID: 25162668<br>PMID: 20130069<br>PMID: 25637076<br>PMID: 24382124<br>PMID: 27613721
Endocrinology	Secondary Hyperparathyroidism	No phosphate binders in patients without dialysis due to lack of evidence regarding clinical endpoints.<br>No routine PTH determination in patients without dialysis; consequently, no calcitriol administration to lower PTH<br>Vitamin D substitution (400 - 800 IU/d) if 25-hydroxyvitamin D [25(OH)D] ≤20 ng/mL<br><br>Denosumab if bone metastases are present	Phosphate binders would mean a very high number of tablets<br><br>Consult nephrologist for dialysis patients	E21.1	UpToDate (18.12.2020)<br>- Management of secondary hyperparathyroidism in adult nondialysis patients with chronic kidney disease<br>- Management of hyperphosphatemia in adults with chronic kidney disease<br><br>PMID: 28646995<br>PMID: 21454719<br>PMID: 21406649
Endocrinology	Vitamin D deficiency (without osteoporosis or bone metastases)	No indication for routine assessment of vitamin D level<br>In patients without osteoporosis and without bone metastases, no indication for calcium administration and/or bone antiresorptive agents<br>Colecalciferol can be considered if vitamin D levels <10 ng/ml.		E55.9	UpToDate (18.12.2020)<br>- Vitamin D deficiency in adults: Definition, clinical manifestations, and treatment<br>- Vitamin D and extraskeletal health<br><br>PMID: 21646368<br>PMID: 25033068<br>PMID: 24729336<br>PMID: 24414552<br>PMID: 29102433

Specialty	Disease	Recommendation for cancer patients with limited life expectancy	Additional information/comment	ICD	Literature
Gastroenterology	Ascites	Therapy depending on cause:<br>- Peritoneal carcinomatosis: only paracentesis, no albumin substitution.<br>- Portal hypertension (also due to hepatic filiae): paracentesis, spironolactone + furosemide 100/40 mg (max 400/160mg); discontinuation of diuretic therapy if ineffective	Paracentesis: Only in case of hepatic cause of ascites, albumin substitution after paracentesis.<br>In malignant ascites, evaluate the implantation of a permanent ascitic drainage system.	R18	UpToDate (28.12.2020)<br>- Evaluation of adults with ascites<br>- Ascites in adults with cirrhosis: Initial therapy<br>- Ascites in adults with cirrhosis: Diuretic-resistant ascites<br>- Malignancy-related ascites<br><br>PMID: 29653741<br>PMID: 23463403<br>PMID: 19700895<br>PMID: 23978348<br>PMID: 22460778
Gastroenterology	Cancer therapy-induced diarrhea	In addition to pausing/discontinuing tumor therapy and initiating symptomatic therapy, the following drugs might be useful: loperamide, tinctura opii, octreotide, racecadotril. Prior to use, exclusion of infectious diarrhea.<br>In the case of immunotherapy (i.e. checkpoint inhibitors) diagnostics with regard to immune-mediated colitis and, if necessary, specific therapy (glucocorticoids, etc.).<br>Discontinuation/change of long-term medication for which diarrhea has been described as a frequent side effect.	Symptomatic therapy includes replacement of nutrients, fluids, electrolytes, vitamins, enteral or parenteral.<br>After radiotherapy, radiation proctitis may also be a cause of diarrhea.	K52.8,<br>K52.9	UpToDate (20.12.2020)<br>- Management of acute chemotherapy-related diarrhea<br>- Enterotoxicity of chemotherapeutic agents<br>- Toxicities associated with checkpoint inhibitor immunotherapy<br><br>PMID: 29931177<br>PMID: 25186048<br>PMID: 30168576<br>PMID: 29177551<br>PMID: 32856210<br>PMID: 29442540
Gastroenterology	Chronic inflammatory bowel diseases: Crohn's disease, ulcerative colitis	No change in therapy in well-controlled disease at the beginning of chemotherapy (exception: steroids). Taper steroid therapy if remission of IBD is stable.<br>If tumor therapy with a known strong immunosuppressive effect is used, it may be possible to switch from oral corticosteroid therapy to budesonide in order to minimize the tendency to infection.<br>Ulcerative colitis: Use of local therapeutics for proctitis or disease confined to the rectum (mesalazine, topical steroid).<br>Discontinuation of probiotics.	In the case of therapy with a biologic agent or combination therapy with azathioprine, colonoscopy control is recommended to confirm histological remission before tapering of drug dosage.	K50.9,<br>K51.9	UpToDate (20.12.2020)<br>- Overview of the medical management of mild (low risk) Crohn disease in adults<br>- Overview of medical management of high-risk, adult patients with moderate to severe Crohn disease<br>- Medical management of low-risk adult patients with mild to moderate ulcerative colitis<br>- Management of moderate to severe ulcerative colitis in adults<br>- Probiotics for gastrointestinal diseases<br><br>PMID: 27660341<br>PMID: 29610508<br>PMID: 30840605<br>PMID: 28870406<br>PMID: 26039678
Gastroenterology	Chronic pancreatitis	General measures (no alcohol, small and low-fat meals, ...)<br>Discontinuation of antioxidants (no effect)<br>Exclude fat malabsorption by determination of pancreatic elastase.<br>Ensure sufficient pancreatic enzyme replacement in exocrine pancreatic insufficiency, i.e. pancreatin up to 80,000 IU per main meal. Addition of a PPI can reduce enzyme inactivation by gastric acid;<br>Attempt of pancreatic enzymes, since they can relieve pain (therapy should be discontinued if it is not effective and there is no exocrine pancreatic insufficiency).<br>Pain therapy: morphine, amitriptyline (Saroten), pregabalin (Lyrica)	Regular evaluation of pancreatic endocrine function to detect insulin deficiency in time and prevent further malnutrition.<br><br>Evaluate pancreatic stenting and stellate ganglion blockade as non-drug measures if pain persists.	K86.0	UpToDate (25.12.2020)<br>- Clinical manifestations and diagnosis of chronic pancreatitis in adults<br>- Treatment of chronic pancreatitis<br><br>PMID: 28344786<br>PMID: 22683257<br>PMID: 25144441<br>PMID: 27941156

Specialty	Disease	Recommendation for cancer patients with limited life expectancy	Additional information/comment	ICD	Literature
Gastroenterology	Diverticulosis	High-fiber diet, sufficient fluid intake, laxatives if necessary, especially in the case of high-emetogenic protocols or concurrent opiate therapy; pain medication as needed, discontinue other medications in this indication.	In case of progressive or new pain, exclude diverticulitis or other causes of pain.	K57	UpToDate (20.12.2020)<br>- Colonic diverticulosis and diverticular disease: Epidemiology, risk factors, and pathogenesis<br>- Royal College of Surgeons (RCS) of England: Commissioning guide – Colonic diverticular disease<br><br>PMID: 21771850<br>PMID: 28494576<br>PMID: 32638537
Gastroenterology	Functional dyspepsia	Attempt to discontinue PPI (if not clearly GERD) and prokinetics (metoclopramide, domperidone) if patient has not experienced clear improvement.<br>History and attempt to discontinue complementary medications.<br>In the case of previously untreated dyspepsia, H.p. eradication if H.p. positive; subsequently, if symptoms persist, therapy trial with PPI and prokinetics	Resolution of symptoms for PPI vs placebo: NNT 15	R10.1	UpToDate (28.12.2020)<br>- Approach to the adult with dyspepsia<br>- Functional dyspepsia in adults<br><br>PMID: 17174612<br>PMID: 25921377<br>PMID: 30335201<br>PMID: 29161458<br>PMID: 28631728
Gastroenterology	Gastric ulcer, duodenal ulcer	Continuation of PPI after initial event in the following situations: Frustrant H.p. eradication, long-term therapy with NSAIDs, elderly comorbid patient with past history of ulcer > 2cm or recurrent ulcers; otherwise PPI can be discontinued after 8-12 weeks.	PPI intake 1 hour before breakfast	K25.9, K26.9	UpToDate (20.12.2020)<br>- Peptic ulcer disease: Treatment and secondary prevention<br>- Approach to refractory peptic ulcer disease<br>- NSAIDs (including aspirin): Treatment of gastroduodenal toxicity<br>- NSAIDs (including aspirin): Primary prevention of gastroduodenal toxicity<br>- NSAIDs (including aspirin): Secondary prevention of gastroduodenal toxicity<br><br>PMID: 28071659<br>PMID: 25954113<br>PMID: 27092708<br>PMID: 28257716
Gastroenterology	Gastroesophageal reflux disease	Continue low-dose PPI therapy in recurrently symptomatic patients, as over 60% of patients experience a recurrence within a few weeks. Attempt to switch to PPI therapy on an as-needed basis possible.<br>Attempt to discontinue drug therapy in patients with a single event in the past (with >8 weeks of therapy; symptomatic or endoscopically confirmed GERD)<br>Maintain or initiate lifestyle modification<br>Avoid NSAIDs	PPI intake 1 hour before breakfast	K21.0, K21.9	UpToDate (20.12.2020)<br>- Clinical manifestations and diagnosis of gastroesophageal reflux in adults<br>- Medical management of gastroesophageal reflux disease in adults<br>- Approach to refractory gastroesophageal reflux disease in adults<br><br>PMID: 28257716<br>PMID: 24789125<br>PMID: 25956834<br>PMID: 23728637

Specialty	Disease	Recommendation for cancer patients with limited life expectancy	Additional information/comment	ICD	Literature
Gastroenterology	Hemorrhoids	In the acute phase, symptomatic therapy with phlebotonics and local therapy is useful.<br><br>Secondary prophylaxis:<br>Attempt to discontinue phlebotonics, i.e. flavonoids<br>A diet rich in fiber is recommended.<br>Evaluate local therapeutic measures such as sclerotherapy or rubber band ligation.		K64	UpToDate (21.12.2020)<br>- Hemorrhoids: Clinical manifestations and diagnosis<br>- Home and office treatment of symptomatic hemorrhoids<br><br>PMID: 29420423<br>PMID: 16235372<br>PMID: 22895941<br>PMID: 16736537<br>PMID: 26309351
Gastroenterology	Helicobacter pylori-gastritis, gastroduodenitis	Discontinuation of PPI therapy after successful H.p. eradication	There is a clear indication for H.p. eradication in ulcers and MALT lymphomas. However, this can also be done if long-term therapy with NSAIDs or aspirin is necessary.	K29.7, K29.9	UpToDate (20.12.2020)<br>- Acute and chronic gastritis due to Helicobacter pylori<br>- Treatment regimens for Helicobacter pylori<br><br>PMID: 28071659<br>PMID: 29089382
Gastroenterology	Liver cirrhosis Child Pugh A/B	Discontinue drugs without proven benefit for the treatment of liver cirrhosis such as Legalon.<br>Hyponatremia: compensate only if neurological symptoms or Na <120 mmol/l, try fluid restriction if Na < 120 mmol/l, compensate for hypokalemia<br><br>Ascites: Discontinue drugs that reduce renal perfusion (NSAIDs, ACE inhibitors/ARBs, ß-blockers), salt restriction, spironolactone/furosemide 100/40 mg (max 400/160 mg), antibiotic prophylaxis after spontaneous bacterial peritonitis (SBP).<br><br>Esophageal varices/portal hypertensive gastropathy: non-selective ß-blocker (primary and secondary prophylaxis). Restraint in ascites/c.n. SBP. For large varices or for secondary prophylaxis, evaluation of endoscopic band ligation.	Beta-blocker to prevent variceal bleeding: NNT 11<br>Avoid tolvaptam because of potential risk of hepatotoxicity	K70.3, 74.6	UpToDate (25.12.2020)<br>- Cirrhosis in adults: Overview of complications, general management, and prognosis<br>- Ascites in adults with cirrhosis: Initial therapy<br>- Primary and pre-primary prophylaxis against variceal hemorrhage in patients with cirrhosis<br>- Prevention of recurrent bleeding from esophageal varices in patients with cirrhosis<br>- Hyponatremia in patients with cirrhosis<br>- Spontaneous bacterial peritonitis in adults: Treatment and prophylaxis<br><br>PMID: 23463403<br>PMID: 25042402<br>PMID: 29653741<br>PMID: 24631577<br>PMID: 10886040<br>PMID: 19277033
Gastroenterology	Non-alcoholic and alcoholic fatty liver disease	No proven drug therapy, therefore discontinuation of all hepatoprotective substances and molecules<br>Recommend lifestyle changes (e.g. Mediterranean diet).		K70.0, K76.0	UpToDate (25.12.2020)<br>- Management of nonalcoholic fatty liver disease in adults<br>- Management of alcohol-associated steatosis and alcohol-associated cirrhosis<br><br>PMID: 28714183<br>PMID: 27062661<br>PMID: 29628280<br>PMID: 29336434<br>PMID: 20578268<br>PMID: 22023985<br>PMID: 15846629<br>PMID: 21678335<br>PMID: 31314133

Specialty	Disease	Recommendation for cancer patients with limited life expectancy	Additional information/comment	ICD	Literature
Gastroenterology	Obstipation	Criteria for constipation present (<3 bowel movements/week, hard stools)?<br>History of medication<br>Step-by-step scheme: general measures (fluids, fiber), macrogol, bisacodyl, bisacodyl clysms, avoid obstipative drugs<br>Painkillers: if opiate is needed, try whether oxycodone hydrochloride/naloxone hydrochloride is better tolerated.	Exclusion of gastrointestinal stenoses especially in GI malignancy/malignancy in the abdominal cavity and after abdominal surgery	K59.0	UpToDate (20.12.2020)<br>- Enterotoxicity of chemotherapeutic agents<br>- Management of chronic constipation in adults<br>- Constipation in the older adult<br>- Prevention and management of side effects in patients receiving opioids for chronic pain<br><br>PMID: 21666546<br>PMID: 27145304<br>PMID: 23261064<br>PMID: 30016389<br>PMID: 23326148<br>PMID: 21440672<br>PMID: 28736104<br>PMID: 21937568
Gastroenterology	Portal hypertension and esophageal varices	see Liver cirrhosis		I85.9, K76.6	
Gastroenterology	Splanchnic vein thrombosis (portal, splenic, mesenteric and supra-hepatic veins)	Preexisting (chronic) or incidental splanchnic vein thrombosis: no anticoagulation except in case of extension of thrombosis.<br>In case of symptomatic (acute) or progressive splanchnic vein thrombosis anticoagulation for at least 6 months as long as the bleeding risk is low. Continuation of anticoagulation (long-term) is recommended in case of malignant splanchnic vein thrombosis, other persistent prothrombotic risk factors or extension of thrombosis.<br><br>In all non-malignant forms of portal vein thrombosis, additionally evaluate TIPS.	Use of DOACs is possible after an initial phase with low molecular weight heparin.	I81	UpToDate (20.12.2020)<br>- Acute portal vein thrombosis in adults: Clinical manifestations, diagnosis, and management<br>- Chronic portal vein thrombosis in adults: Clinical manifestations, diagnosis, and management<br><br>PMID: 26516032<br>PMID: 19399912<br>PMID: 19081529<br>PMID: 17958760<br>PMID: 28479379<br>PMID: 31895720<br>PMID: 29063233<br>PMID: 32078681<br>PMID: 31293916
Gynecology	Endometriosis	At the onset of menopause under chemotherapy (laboratory criteria: FSH 20-100 IU/ml, LH >30 IU/ml, estradiol <30 µg/ml) discontinuation of endocrine therapy	Endometriosis can also cause symptoms postmenopausally	N80.9	UpToDate (28.12.2020)<br>- Endometriosis: Treatment of pelvic pain<br>- Endometriosis of the bladder and ureter<br>- Endometriosis: Treatment of rectovaginal and bowel disease<br>- Endometriosis: Long-term treatment with gonadotropin-releasing hormone agonists<br><br>PMID: 28114727<br>PMID: 25644508<br>PMID: 22646295<br>PMID: 23528916<br>PMID: 24630080

Specialty	Disease	Recommendation for cancer patients with limited life expectancy	Additional information/comment	ICD	Literature
Hematology	Anemia in chronic kidney disease	Iron deficiency must be corrected before starting ESA therapy.<br>Therapy goal of ESA therapy: Hb 10 - 11.5 G/dl	Intravenous iron substitution during ESA therapy (see also iron deficiency anemia)	D63.8	UpToDate (18.12.2020)<br>- Treatment of anemia in nondialysis chronic kidney disease<br>- Treatment of iron deficiency in nondialysis chronic kidney disease (CKD) patients<br>- Treatment of anemia in hemodialysis patients<br><br>KDIGO Clinical Practice Guideline for Anemia in Chronic Kidney Disease https://kdigo.org/wp-content/uploads/2016/10/KDIGO-2012-Anemia-Guideline-English.pdf<br><br>PMID: 23891356<br>PMID: 19037082<br>PMID: 28066881
Hematology	Anemia of chronic disease/inflammation including cancer	Treatment indication in case of progressive severe anemia (Hb drop <8 g/dl) and/or symptoms of anemia<br>Principle Goal of ESA therapy: Dose adjustments to avoid transfusion and symptoms of anemia (see also iron deficiency anemia)<br>Dose reduction as soon as therapy goal is reached or Hb increase >1 g/dL within 2 weeks after start of therapy.<br>Discontinuation if Hb >12 g/dL or in case of lack of response despite adequate dose increase and iv iron substitution (see iron deficiency anemia) after 8 weeks	Iron deficiency must be corrected before starting therapy.	D64.9	UpToDate (18.12.2020)<br>- Hematologic complications of malignancy: Anemia and bleeding<br>- Role of erythropoiesis-stimulating agents in the treatment of anemia in patients with cancer<br><br>https://www.nccn.org/ (18.12.2020)<br>- NCCN Guidelines: Hematopoietic Growth Factors, Version 2.2020<br>(including Management of Cancer- and Chemotherapy-Induced Anemia)<br><br>https://www.onkopedia.com/de/ (18.12.2020)<br>- Eisenmangel und Eisenmangelanämie<br><br>PMID: 22877242<br>PMID: 20974674<br>PMID: 23235597<br>PMID: 18227526<br>PMID: 20555090<br>PMID: 30285221<br>PMID: 26373748
Hematology	Folic acid deficiency anemia	Folic acid substitution (0.4 mg/d or 1x 5mg/week until normalization, then discontinuation attempt)	Folic acid determination always at the same time as Vit B12 determination	D52.9	UpToDate (18.12.2020)<br>- Clinical manifestations and diagnosis of vitamin B12 and folate deficiency<br>- Treatment of vitamin B12 and folate deficiencies<br><br>PMID: 24942828

Specialty	Disease	Recommendation for cancer patients with limited life expectancy	Additional information/comment	ICD	Literature
Hematology	Iron deficiency anemia	Principle Goal: Avoid transfusion and symptoms of anemia<br>An already ongoing oral iron therapy can be discontinued if Hb >11 g/dl and previously decreased ferritin and transferrin saturation are normalized.<br>If iron deficiency is diagnosed at baseline or during the course of the disease, iv iron substitution is recommended.<br>Intravenous iron substitution if Hb <11g/dL and<br>- ferritin < 30 ng/mL and/or transferrin saturation <20% OR<br>- absence of Hb increase under ESA treatment with ferritin 30-500 (-800) ng/mL and transferrin saturation <50%<br>If iron deficiency persists and Hb <11 g/dl despite adequate oral therapy, a switch to iv therapy is advisable (better efficacy).	Since ferritin is often elevated in chronic inflammation, it is advisable to also determine the transferrin saturation in order not to overlook an iron deficiency.	D50.9	UpToDate (17.12.2020)<br>- Treatment of iron deficiency anemia in adults<br>- Evaluation and management of anemia and iron deficiency in adults with heart failure<br>- Role of erythropoiesis-stimulating agents in the treatment of anemia in patients with cancer<br><br>https://www.nccn.org/ (17.12.2020)<br>NCCN Guidelines: Hematopoietic Growth Factors, Version 2.2020<br>(including Management of Cancer- and Chemotherapy-Induced Anemia)<br><br>https://www.onkopedia.com/de/ (17.12.2020)<br>- Eisenmangel und Eisenmangelanämie<br><br>PMID: 22877242<br>PMID: 23983177<br>PMID: 30285221<br>PMID: 26373748
Hematology	Vitamin B12 deficiency anemia	1000 µg Vit B12 parenterally (Cyanocobalamin or Hydroxocobalamin iv in short infusion or im) at least once per week until Vit B12 normalizes, then once per month or every other month;<br>Discontinuation or prolongation of the application intervals makes sense if the Vit B12 level is normal or normalized (Vit B12 control after 3 and 12 months)		D51.9	UpToDate (18.12.2020)<br>- Clinical manifestations and diagnosis of vitamin B12 and folate deficiency<br>- Treatment of vitamin B12 and folate deficiencies<br><br>PMID: 24942828<br>PMID: 29543316<br>PMID: 23301732
Infectious Disease	Hepatitis B	Therapy for recent infection according to guidelines.<br><br>Drug prophylaxis of HBV reactivation in solid tumors is indicated in "moderate to very high risk", i.e. in patients with positive HBsAg and either anti-CD20 therapy, high-dose glucocorticoids (>20 mg/day prednisolone), cytotoxic chemotherapy, anti-TNF therapy; in the case of positive anti-HBc but negative HBsAg and negative PCR, regular PCR progress controls in solid tumors; preferred drugs tenofovir, entecavir<br><br>Vaccination in case of negative serology	HBV reactivation in HBV carriers under chemotherapy 14-72%<br>Mortality due to HBV reactivation in this population 5-52%<br><br>Diagnosis by means of hepatitis serology and PCR (risk of reactivation with positive HbsAg or positive anti-Hbc)	B19.9	UpToDate (17.12.2020)<br>- Hepatitis B virus: Screening and diagnosis<br>- Hepatitis B virus: Overview of management<br>- Hepatitis B virus reactivation associated with immunosuppressive therapy<br><br>PMID: 25447850<br>PMID: 28427875<br>PMID: 26595058<br>PMID: 22271089<br>PMID: 29858430<br>PMID: 33067316

Specialty	Disease	Recommendation for cancer patients with limited life expectancy	Additional information/comment	ICD	Literature
Nephrology	Chronic renal failure (Chronic kidney disease, CKD)	Avoid progression (exclude urinary tract obstruction, discontinue nephrotoxic substances, regularly review choice and dose of chemotherapeutic agents)<br>No dietary measures<br>Reduce/discontinue antihypertensives in asymptomatic hypertension (upper limit: systolic blood pressure 140 mmHg).<br>Statin only in cases of confirmed atherosclerosis<br>No clear recommendation of phosphate binders, thus discontinuation possible<br>Metabolic acidosis: NaBic 0.5 to 1 mEq/kg/d<br>Secondary HPT: see chapter "Secondary hyperparathyroidism"		N18.1-N18.5	UpToDate (27.12.2020)<br>- Overview of the management of chronic kidney disease in adults<br>- Dietary recommendations for patients with nondialysis chronic kidney disease<br>- Pathogenesis, consequences, and treatment of metabolic acidosis in chronic kidney disease<br>- Management of hyperphosphatemia in adults with chronic kidney disease<br>- Overview of kidney disease in the cancer patient<br><br>https://kdigo.org/<br>(27.12.2020)<br>- KDIGO: Clinical practice guideline for the evaluation and management of chronic kidney disease, 2012<br><br>PMID: 27807144<br>PMID: 28288249<br>PMID: 17634949
Nephrology	Nephrotic syndrome	Medical history: pre-existing nephrotic syndrome or due to tumor disease? (membranous GN improves with successful tumor treatment)<br>Avoid progression (discontinue nephrotoxic substances, regularly review choice and dose of chemotherapeutic agents).<br>Stricter adjustment of blood pressure is useful to reduce proteinuria (systol. approx. 125-130mmHg); adjustment with ATII blocker or ACE inhibitor<br>Continue steroid/IS according to the cause of GN/proteinuria<br>Peripheral oedema: Salt restriction (<2 g/d) and loop diuretic<br>Statin in case of confirmed atherosclerosis and hyperlipidemia<br>Prophylactic anticoagulation in membranous GN and serum albumin < 3g/l and low risk of bleeding.		N04	UpToDate (26.12.2020)<br>- Overview of heavy proteinuria and the nephrotic syndrome<br>- Pathophysiology and treatment of edema in adults with the nephrotic syndrome<br>- Hypercoagulability in nephrotic syndrome<br>- Lipid abnormalities in nephrotic syndrome<br><br>PMID: 30665568<br>PMID: 30665569<br>PMID: 23871408<br>PMID: 24327265<br>PMID: 24336031<br>PMID: 28288249<br>PMID: 21372254
Neurology	Dementia	Change of therapy only in consultation with the psychiatrist/neurologist in charge.<br>Alzheimer's dementia: Continuation of current therapy with cholinesterase inhibitor and/or memantine with proven positive effect on cognition and activities of daily living and few side effects.<br>In dementia associated with idiopathic Parkinson's disease: continuation of current therapy with rivastigmine with proven positive effect on cognition and activities of daily living and few side effects.<br><br>Discontinuation of gingko preparations, statins, omega-3 fatty acids, vitamin B, vitamin E, selegiline	Alzheimer's dementia: memantine (NNT ~10), acetylcholinesterase inhibitors (NNT ~7)<br><br>The usefulness of tumor therapy for moderate/severe dementia must be assessed on an individual basis.<br>Mini-Mental State Examination (MMSE)<br>Mild: >19<br>Moderate: 10-19<br>Severe: <10	F01, F02	UpToDate (18.12.2020)<br>- Treatment of dementia<br>- Cholinesterase inhibitors in the treatment of Alzheimer disease<br><br>PMID: 20831773<br>PMID: 30011160<br>PMID: 27063583<br>PMID: 19160216<br>PMID: 28128435<br>PMID: 16437532<br>PMID: 29923184<br>PMID: 31638686<br>PMID: 12535396<br>PMID: 25808982

Specialty	Disease	Recommendation for cancer patients with limited life expectancy	Additional information/comment	ICD	Literature
Neurology	Epilepsy	Consultation with the neurologist in charge in case of potential interactions/side-effects with planned tumor therapy and follow-up during therapy		G40.9	UpToDate (19.12.2020)<br>- Overview of the management of epilepsy in adults<br>- Initial treatment of epilepsy in adults<br>- Seizures and epilepsy in older adults: Treatment and prognosis<br><br>PMID: 29898971<br>PMID: 29898974
Neurology	Ischemic stroke or transient ischemic attack due to cerebrovascular disease	see "Confirmed atherosclerotic cardiovascular disease (CVD) including coronary artery disease (CAD), cerebrovascular disease (CVD) and peripheral artery disease (PAD)"		I63.9, I67.2, G45.9	UpToDate (20.12.2020)<br>- Antiplatelet therapy for the secondary prevention of ischemic stroke<br>- Overview of secondary prevention of ischemic stroke<br><br>PMID: 29633334<br>PMID: 28528288
Neurology	Migraine	Consultation with the neurologist in charge in case of potential interactions/side-effects with planned tumor therapy and follow-up during therapy.<br><br>Continue effective secondary drug prophylaxis in case of more than one attack per month. If migraine worsens during tumor therapy, start or change drug therapy.<br><br>Acute therapy of migraine: NSAIDs, especially naproxen, triptans (e.g. sumatriptan)		G43.9	UpToDate (19.12.2020)<br>- Acute treatment of migraine in adults<br>- Preventive treatment of episodic migraine in adults<br><br>DGN Leitlinie 2018: Therapie der Migräneattacke und Prophylaxe der Migräne<br><br>PMID: 22529202<br>PMID: 19708964<br>PMID: 23592242<br>PMID: 27351677<br>PMID: 25877672
Neurology	Multiple sclerosis	Consultation with the neurologist in charge in case of potential interactions/side-effects with planned tumor therapy and follow-up during therapy		G35.9	UpToDate (19.12.2020)<br>- Symptom management of multiple sclerosis in adults<br>- Disease-modifying therapies for multiple sclerosis: Pharmacology, administration, and adverse effects<br><br>PMID: 29686116<br>PMID: 32781983<br>PMID: 28870581<br>PMID: 29353550
Neurology	Parkinson's syndrome	Consultation with the neurologist in charge of the patient with regard to potential interactions/side-effects with planned tumor therapy and follow-up during therapy		G20.9, G21.9	UpToDate (19.12.2020)<br>- Initial pharmacologic treatment of Parkinson disease<br>- Palliative approach to Parkinson disease and parkinsonian disorders<br><br>PMID: 30653247<br>PMID: 29570866

**Specialty**	**Disease**	**Recommendation for cancer patients with limited life expectancy**	**Additional information/comment**	**ICD**	**Literature**
Neurology	Polyneuropathy	Continue ongoing effective therapy for preexisting polyneuropathy.<br>No established drug therapy for primary prevention of chemotherapy-induced peripheral neuropathy (CIPN)<br><br>Symptom-oriented therapy decisions: both dose increase and reduction attempt possible (discontinuation in case of lack of efficacy at maximum doses); Consider combination therapy with opiates<br>Combine additional physical measures, topical therapy or cognitive therapies	Typical drugs are duloxetine, gabapentin, pregabalin, amitryptiline.<br>For chemotherapy-induced painful PNP: 1st choice duloxetine (moderate evidence).	G62.0, G62.9	UpToDate (19.12.2020)<br>- Overview of polyneuropathy<br>- Management of diabetic neuropathy<br>- Prevention and treatment of chemotherapy-induced peripheral neuropathy<br>PMID: 34226683<br>PMID: 32663120<br>PMID: 21482920<br>PMID: 30052758<br>PMID: 20402746<br>PMID: 23549581<br>PMID: 24385423<br>PMID: 18356405
Neurology	Psychiatric disorders with medication therapy setting (Bipolar affective disorder, Depressive episode, Recurrent depressive disorder, Anxiety disorder, Obsessive-compulsive disorder, Adjustment disorder, Non-organic sleep disorder).	Change of therapy only in consultation with neurologist/psychiatrist with regard to necessity of therapy and potential interactions/side effects with planned tumor therapy (i.e.serotonin syndrome due to SSRI, 5HT3 Antagonists, tramadol,...)<br>Treatment of depression is useful even if life expectancy is short.	Unipolar depression: therapy should be continued for at least 4-9 months beyond the remission of the depressive episode. After several episodes or episodes with significant functional limitations, long-term prophylaxis should be given for at least 2 years.<br><br>Benefits of antidepressant therapy depending on severity:<br>Mild depressive episode NNT ~16<br>Moderate depressive episode NNT ~11<br>Major depressive episode NNT ~4	F31.9, F32.9, F33.9, F41.9, F42.9, F43.2, F51.9	UpToDate (19.12.2020)<br>- Clinical features, assessment, and diagnosis of unipolar depressive disorders in patients with cancer<br>- Management of psychiatric disorders in patients with cancer<br><br>S3 Leitlinie unipolare Depression, 2017<br><br>PMID: 20051569<br>PMID: 24716498<br>PMID: 29683474
Neurology	Vertigo	Attempt to discontinue drug therapies (e.g. dimenhydrinate) in absence of clinical improvement.<br>Complementary vestibular physiotherapy can be helpful.		R42	UpToDate (25.12.2020)<br>- Evaluation of the patient with vertigo<br>- Treatment of vertigo<br><br>PMID: 17943853<br>PMID: 27327415<br>PMID: 25485940<br>PMID: 26804483
Ophthalmology	Glaucoma	Continue local therapy	Ophthalmological check-up in case of glucocorticoid therapy >2 weeks	H40.9	UpToDate (19.12.2020)<br>- Open-angle glaucoma: Treatment<br><br>PMID: 29106798
Ophthalmology	Wet macular degeneration	No change in follow-up and/or ongoing therapy		H35.30	UpToDate (28.12.2020)<br>- Age-related macular degeneration: Treatment and prevention<br><br>PMID: 30303083<br>PMID: 28686003<br>PMID: 28860733

Specialty	Disease	Recommendation for cancer patients with limited life expectancy	Additional information/comment	ICD	Literature
Orthopedics	Arthrosis (polyarthrosis, coxarthrosis, gonarthrosis, arthrosis of the spinal column, etc.), spondylosis	Maintain or initiate non-pharmacological therapy (physiotherapy/physical activity).<br>Therapy attempt with topical NSAIDs possible (exceptions: polyarthrosis, coxarthrosis); capsaicin patches.<br>Oral NSAIDs at the lowest possible dose, combination with PPI in patients with comorbidities such as diabetes, hypertension or advanced age<br>Opiates and paracetamol often not very effective (therapy trial possible, discontinue if ineffective)<br>Discontinuation of food supplements such as glucosamine, chondroitin, vitamin D, diacerein, avocado soybean oil and fish oil	Pain reduction through topical diclofenac (especially in gonarthrosis): NNT 9.8<br>Pain reduction and improvement of mobility through physical training in coxarthrosis: NNT 6	M15.9, M16.9, M17.9, M19.99, M47.99	UpToDate (26.12.2020)<br>- Overview of the management of osteoarthritis<br>- Management of knee osteoarthritis<br>- Comorbidities that impact management of osteoarthritis<br>- Management of hip osteoarthritis<br><br>PMID: 31278997<br>PMID: 31908149<br>PMID: 27103611<br>PMID: 25828856<br>PMID: 29018060<br>PMID: 24756895<br>PMID: 26405113<br>PMID: 30154087
Orthopedics	Chondrocalcinosis, pseudogout, Calcium Pyrophosphate Deposition	In acute chondrocalcinosis, NSAIDs, colchicine (low-dose) or prednisolone (analogous to acute arthritis urica). Intra-articular corticosteroid injection possible in the absence of contraindications.<br>Evaluate colchicine prophylaxis in frequent acute attacks (>3 times/year), taking into account possible side effects.	Incidence of radiographic chondrocalcinosis: 65-74 years: 15%; 75-84 years: 36% and >84 years: 50%. Most patients are asymptomatic. In acute CPPD, the knee joint is affected in 50% of cases (then wrist, elbow, shoulder joint). Iron overload is a risk factor for chondrocalcinosis. Diagnosis and therapy of possible causes/concomitant diseases such as haemochromatosis, hyperparathyroidism, hypomagnesaemia, hypophosphatemia, etc.	M11.1, M11.2	UpToDate (26.12.2020)<br>- Pathogenesis and etiology of calcium pyrophosphate crystal deposition (CPPD) disease<br>- Clinical manifestations and diagnosis of calcium pyrophosphate crystal deposition (CPPD) disease<br>- Treatment of calcium pyrophosphate crystal deposition (CPPD) disease<br><br>PMID: 21257614<br>PMID: 28328803<br>PMID: 30986010
Orthopedics	Subacute and chronic low back pain	Physical therapy, psychological interventions (behavioral therapy) according to international recommendations.<br>Discontinuation of dietary supplements such as glucosamine, herbal medicine or anti-TNF alpha therapy (Infliximab)<br>Oral NSAIDs at the lowest possible dose for symptom relief (paracetamol often not very effective). Cox2 inhibitors or non-selective NSAIDs with PPI in patients with comorbidities.<br>If not sufficient, additional muscle relaxant (cautious use in elderly patients), e.g. tizanidine.<br>If not effective, attempt tramadol or duloxetine.<br>Opiates only if response to the above options is insufficient (higher rate of side effects than non-opiate therapy)	In case of new or increasing complaints, clarification of osseous metastases is recommended	M54.5	UpToDate (26.12.2020)<br>- Subacute and chronic low back pain: Nonpharmacologic and pharmacologic treatment<br>- Exercise-based therapy for low back pain<br>- Treatment of persistent pain in older adults<br>- Pharmacologic management of chronic non-cancer pain in adults<br>- Complementary and alternative remedies for rheumatic disorders<br><br>PMID: 28192789<br>PMID: 25681408<br>PMID: 27271789<br>PMID: 26863524<br>PMID: 20606148
Orthopedics	Tendinitis calcarea in the shoulder area	Physical therapy<br>Discontinuation of dietary supplements such as glucosamine, herbal medicine.<br>Oral NSAIDs at the lowest possible dose for symptom relief. Cox2 inhibitors or non-selective NSAIDs with PPI in patients with comorbidities.<br>Glucocorticoid injection only in acute attacks, not for long-term therapy	Often spontaneous improvement within 3-6 months. If symptoms persist, try adjunct extracorporeal shock wave therapy	M75.3	UpToDate (26.12.2020)<br>- Calcific tendinopathy of the shoulder<br>- Evaluation of the adult with shoulder complaints<br>- Basic calcium phosphate (BCP) crystal arthritis, including Milwaukee shoulder syndrome<br><br>PMID: 24733195<br>PMID: 19790063

**Specialty**	**Disease**	**Recommendation for cancer patients with limited life expectancy**	**Additional information/comment**	**ICD**	**Literature**
Otolaryngology	Tinnitus	Discontinue drug therapy (exceptions can be antidepressants and sedatives, as depression and insomnia can intensify tinnitus!)<br>Eliminate triggering causes (cerumen, serotympanum)<br>Hearing examination and, if necessary, prescription of hearing aids	Ginko supplements, zinc and other food supplements have no proven benefit	H93.1	UpToDate (28.12.2020)<br>- Treatment of tinnitus<br>- Clinical use of ginkgo biloba<br><br>PMID: 29601255<br>PMID: 25273878<br>PMID: 27995315
Psychiatry	Delirium	Change of therapy with regard to necessity of therapy and potential interactions/side effects with planned tumor therapy	Early reduction of polypharmacy and drugs with anticholinergic burden. Avoid onset of benzodiazepines for patients at risk for delirium/during delirium. Non-medical interventions for delirium patients according to guidelines.	F05	UpToDate (10.11.2021)<br>- Delirium and acute confusional states: Prevention, treatment, and prognosis<br><br>PMID: 29992308<br>PMID: 28187050
Pulmonology	Asthma	Medical history for classification and severity of the disease<br>Escalation and de-escalation according to a step-by-step scheme:<br>Stage 1: low dose inhaled corticosteroid-formoterol on demand<br>Step 2: low dose inhaled corticosteroids-formoterol 2x daily<br>Stage 3: low dose inhaled corticosteroids-LABA<br>Stage 4: medium dose inhaled corticosteroids-LABA<br>Stage 5: high dose inhaled corticosteroids-LABA, additional drug therapies after consultation with the treating pulmonary specialist	Chemotherapy and concomitant medication can reduce symptoms and the need for therapy<br><br>As a simplification of the step-by-step scheme, inhaled corticosteroids-formoterol is recommended both as a basic therapy and as a supplementary medication on demand (SMART concept)	J45.9	UpToDate (20.12.2020)<br>- An overview of asthma management<br>- Acute exacerbations of asthma in adults: Home and office management<br>- Treatment of intermittent and mild persistent asthma in adolescents and adults<br>- Treatment of moderate persistent asthma in adolescents and adults<br>- Treatment of severe asthma in adolescents and adults<br><br>GINA Guidelines 2020<br><br>PMID: 26206872<br>PMID: 30521673<br>PMID: 29938789<br>PMID: 27582089<br>PMID: 28933516<br>PMID: 28301050<br>PMID: 15266477
Pulmonology	Chronic cough (without local cause)	Eliminate potential triggers: ACE inhibitors, ATII blockers, GERD, asthma, COPD.<br>Discontinue non-specific antitussives (dextromethorphan, codeine, ...)	Non-pharmacological therapies (speech therapy, breathing exercises and cough suppression techniques) may be tried.	R05	UpToDate (25.12.2020)<br>- Evaluation of subacute and chronic cough in adults<br>- Treatment of subacute and chronic cough in adults<br><br>PMID: 26426314<br>PMID: 23928798<br>PMID: 24121952<br>PMID: 25989380

Specialty	Disease	Recommendation for cancer patients with limited life expectancy	Additional information/comment	ICD	Literature
Pulmonology	Chronic obstructive pulmonary disease (COPD)	Staged regimen according to GOLD classification<br>A: Inhaled short-acting beta-agonists (SABA) or anticholinergics (SAMA) if needed.<br>B: Long-acting beta-agonists (LABA) or anticholinergics (LAMA), with additional SABA or SAMA if needed.<br>C: LAMA, if necessary additionally SABA<br>D: LAMA and LABA, if necessary add inhaled corticosteroid (ICS)<br>Phosphodiesterase-4 inhibitors only for category D<br>Inhaled glucocorticosteroids should be avoided because of the tendency to infection.<br><br>Attempt to discontinue mucolytics and theophylline<br>Vaccinations recommended (pneumococcus, influenza)<br><br>Azithromycin prophylaxis only for very frequent exacerbations of infections; discontinue azithromycin if exacerbations do not improve (after consultation with treating pulmonary specialist)	Chemotherapy and concomitant medication can reduce symptoms and the need for therapy	J44.99, J44.90, J44.91, J44.92, J44.93, J45.9	UpToDate (20.12.2020)<br>- Chronic obstructive pulmonary disease: Definition, clinical manifestations, diagnosis, and staging<br>- Stable COPD: Initial pharmacologic management<br>- Management of refractory chronic obstructive pulmonary disease<br>- COPD exacerbations: Management<br>- Management of infection in exacerbations of chronic obstructive pulmonary disease<br><br>PMID: 26222376<br>PMID: 30326124<br>PMID: 16437444<br>PMID: 28116747<br>PMID: 30376188<br>PMID: 30846476<br>PMID: 28298398
Pulmonology	Interstitial lung disease and pulmonary fibrosis	Pneumococcal and influenza vaccination<br>Idiopathic pulmonary fibrosis: weighing benefits/risks of nintedanib and pirfenidone due to P-GP and CYP3A4 interactions;<br>Attempt to discontinue if side effects occur that may only occur or be exacerbated with chemotherapy, such as diarrhea (62%), nausea (24%), vomiting (12%), or increase in transaminases (14%)<br>Discontinuation of ineffective drugs (e.g. acetylcysteine, various immunosuppressants, anticoagulation, ...)<br>Steroid reduction possible in long-term therapy (due to immunosuppression under chemotherapy and concomitant medication)		J84.9	UpToDate (20.12.2020)<br>- Treatment of idiopathic pulmonary fibrosis<br>- Acute exacerbations of idiopathic pulmonary fibrosis<br><br>PMID: 26177183<br>PMID: 26915984<br>PMID: 27876247<br>PMID: 22607134<br>PMID: 31112379
Pulmonology	Sarcoidosis	Adjust ongoing glucocorticoid therapy according to oncological therapy (usually glucocorticoid-containing); maintain control intervals, consult with treating specialist.		D86.9	UpToDate (18.12.2020)<br>- Treatment of pulmonary sarcoidosis: Initial therapy with glucocorticoids<br><br>PMID: 29719195<br>PMID: 32293205
Rheumatology	Fibromyalgia	Patient education and exercise therapy, drug therapy if unsuccessful.<br>Maintaining effective pharmacological therapy, e.g. with amitriptyline, duloxetine, milnacipran or pregabalin.<br>Changeover if ineffective	NNT for 30% pain reduction:<br>Amitriptyline 3.5<br>Duloxetine 8.2<br>Milnacipran 10.9<br>NNT 7-14 for different levels of pain reduction or different doses of pregabalin (dose 300-600mg/d).<br><br>Overall, poor efficacy of drug therapy on fatigue and sleep disturbances	M79.90	UpToDate (26.12.2020)<br>- Initial treatment of fibromyalgia in adults<br>- Treatment of fibromyalgia in adults not responsive to initial therapies<br><br>PMID: 27377815<br>PMID: 21078630<br>PMID: 24385423<br>PMID: 29489029<br>PMID: 27684492<br>PMID: 24362925<br>PMID: 20459730<br>PMID: 28636204

Specialty	Disease	Recommendation for cancer patients with limited life expectancy	Additional information/comment	ICD	Literature
Rheumatology	Giant cell arteritis (GCA, temporal arteritis)	Initiation and/or continuation of a guideline-based therapy (glucocorticoids, tolicizumab).<br>Maintain regular follow-up. Due to the immunosuppressive effect of tumor therapy, attempt to reduce ongoing steroid therapy by 5 mg decrements for long-term prednisone ≥20 mg.<br>Careful clinical follow-up and regular determination of the erythrocyte sedimentation rate and the C-reactive protein.<br>Low-dose aspirin and statin therapy in the presence of atherosclerosis.	Routine prophylaxis against Pneumocystis jirovecii pneumonia (PCP) only in severe cases with long-term prednisone doses ≥20 mg.	M31.5, M31.6	UpToDate (28.12.2020)<br>- Treatment of giant cell arteritis<br>- Clinical manifestations of giant cell arteritis<br><br>PMID: 26359488<br>PMID: 20371504<br>PMID: 27299619<br>PMID: 28905861
Rheumatology	Polymyalgia rheumatica	Initiation and/or continuation of a guideline-based glucocorticoid therapy.<br>Due to the immunosuppressive effect of tumor therapy, attempt to reduce ongoing steroid therapy by 2.5 mg decrements for long-term prednisone ≥15 mg when no symptoms are present.<br>Careful clinical follow-up and regular determination of the erythrocyte sedimentation rate and the C-reactive protein.	Consideration of the amount of steroids in the context of chemotherapy. Frequent relapses if steroid taper is too fast<br><br>Routine prophylaxis against Pneumocystis jirovecii pneumonia (PCP) only in severe cases with long-term prednisone doses ≥20 mg	M35.3	UpToDate (28.12.2020)<br>- Clinical manifestations and diagnosis of polymyalgia rheumatica<br>- Treatment of polymyalgia rheumatica<br><br>PMID: 26359488<br>PMID: 19910443<br>PMID: 27299619<br>PMID: 28905861<br>PMID: 28460395<br>PMID: 31493201
Rheumatology	Reactive Arthritis	Attempt to discontinue NSAIDs in case of long-term diagnosis and lack of complaints. No indication for long-term glucocorticoid therapy. Attempt to discontinue disease-modifying anti-rheumatic drugs (DMARDs) if ineffective based on history.<br>Antibiotic therapy not indicated by default (exception: active infection with Chlamydia trachomatis)	Infectious enteritis and urinary tract infections are possible triggers (Chlamydia trachomatis, Yersinia, Salmonella, Shigella, Campylobacter, Clostridium difficile, Chlamydia pneumoniae), otherwise reactive arthritis is a diagnosis of exclusion.<br>In case of persistence >12 months, re-evaluate the diagnosis	M02.9	UpToDate (25.12.2020)<br>- Reactive arthritis<br>- Clinical manifestations and diagnosis of Chlamydia trachomatis infections<br><br>PMID: 23588936<br>PMID: 29455267<br>PMID: 28087505
Rheumatology	Rheumatoid arthritis	Regular assessment of symptoms (goal: freedom from symptoms).<br>Due to the immunosuppressive effect of tumor therapy, ongoing steroid or NSAID therapy can often be reduced, as can disease-modifying anti-rheumatic drug (DMARD) therapy, especially biologics (additional risk of infection).	NSAIDs and glucocorticoids should only be used in RA to bridge the gap until DMARD therapy is sufficiently effective	M05.99<br>M06.09<br>M06.99	UpToDate (25.12.2020)<br>- General principles of management of rheumatoid arthritis in adults<br>- Initial treatment of rheumatoid arthritis in adults<br>- Use of glucocorticoids in the treatment of rheumatoid arthritis<br><br>PMID: 31969328<br>PMID: 26545825<br>PMID: 27979873<br>PMID: 28264816<br>PMID: 20671022

Specialty	Disease	Recommendation for cancer patients with limited life expectancy	Additional information/comment	ICD	Literature
Urology	Benign prostate hyperplasia	Therapy only in case of obstruction-related, disturbed bladder emptying or restriction of quality of life; alpha-1 blockers are the therapy of choice; 5-alpha-reductase inhibitors in case of intolerance (onset of action after 6-12 months); anticholinergics in case of irritative symptoms (hyperactive bladder); phosphodiesterase-5 inhibitors in case of additionally existing erectile dysfunction; no phytotherapeutics	Alpha-1 blocker vs placebo within 4 years: NNT 19 (3 years) to avoid clinical progression<br>Finasteride vs placebo: surgery for BPH 5% VS 10% -> NNT 20 (4 years) -> NNT 27 (3 years) to avoid surgery; NNT 33 (3 years) for prevention of acute urinary retention	N40	UpToDate (28.12.2020)<br>- Clinical manifestations and diagnostic evaluation of benign prostatic hyperplasia<br>- Medical treatment of benign prostatic hyperplasia<br><br>PMID: 20944799<br>PMID: 27717522<br>PMID: 15610102<br>PMID: 20927745<br>PMID: 9475762<br>PMID: 30480763<br>PMID: 23235581<br>PMID: 30332601
Urology	Erectile dysfunction	Attempt to discontinue antidepressants or change therapy only after consultation with the psychiatrist in charge.<br>Attempt to discontinue thiazides or spironolactone after checking the indication and weighing the necessity of diuretic therapy.<br>Attempt testosterone substitution only in cases of clear deficiency<br>First-line therapy: phophodiesterase-5 inhibitors<br>The use of potency-enhancing food supplements is not recommended.	Successful sexual intercourse: PDE-5 inhibitors 69% vs. placebo 35%<br>Improvement in erections: PDE-5 inhibitors 67%-89% vs. placebo 27%-35%<br>Phophodiesterase-5 inhibitors: Note contraindications, especially nitrate therapy in heart disease<br>Phophodiesterase-5 inhibitors in combination with alpha-blockers may cause hypotension.	N48, F52	UpToDate (28.12.2020)<br>- Treatment of male sexual dysfunction<br>- Sexual activity in patients with cardiovascular disease<br><br>PMID: 29227723<br>PMID: 29746858<br>PMID: 28923561<br>PMID: 29550461<br>PMID: 12076233<br>PMID: 19884626
Urology	Interstitial cystitis/bladder pain syndrome	Attempt to discontinue in the absence of clinical improvement.<br>Consider trying therapy with amitryptiline or pentosan polysulphate for symptomatic pain reduction.<br>Analgesics (NSAIDs, e.g. ibuprofen) may be used for short-term relief for flares of bladder pain.		N30	UpToDate (27.12.2020)<br>- Interstitial cystitis/bladder pain syndrome: Clinical features and diagnosis<br>- Management of interstitial cystitis/bladder pain syndrome<br><br>PMID: 26272202<br>PMID: 25623737<br>PMID: 30566978
Urology	Recurrent urinary tract infections (after exclusion of a locally correctable cause)	Stop prophylaxis with cranberry juice, phenazopyridines, D-mannose, especially if there is no improvement.<br>Short term antibiotic therapy according to antibiogram susceptibility (urine culture)<br>Long-term antibiotic prevention only after detailed clarification for a period of 3-6 months, re-evaluation after 3 months: discontinue long-term antibiotic treatment in the absence of clinical improvement.<br>Attempt vaginally administered estrogens in postmenopausal women (after gynecological assessment and absence of contraindications concerning the tumor disease)	NNT 1.9 to prevent clinical relapse with long-term antibiotic treatment, but also side effects<br><br>Contradictory data to cranberry products with respect to relapse prophylaxis. Thus, no routine recommendation, therapy trial possible if patient wishes.<br><br>Clarification of treatable causes and detailed counselling of the patient to avoid risky behavior before starting long-term antibiotic treatment.	N39	UpToDate (28.12.2020)<br>- Recurrent simple cystitis in women<br><br>https://uroweb.org/wp-content/uploads/EAU-Guidelines-on-Urological-Infections-2018-large-text.pdf (30.09.2019)<br>- European Association of Urology (EAU): Urological infections guidelines<br><br>https://www.awmf.org/uploads/tx_szleitlinien/043-044l_S3_Harnwegsinfektionen_2017-05.pdf (28.12.2020)<br><br>PMID: 22031610<br>PMID: 15266443<br>PMID: 18425910<br>PMID: 23076891<br>PMID: 31042112

Specialty	Disease	Recommendation for cancer patients with limited life expectancy	Additional information/comment	ICD	Literature
Urology	Urinary incontinence	Maintain or initiate non-pharmacological therapy (pelvic floor training, biofeedback method, ...)<br>Attempt to discontinue/switch medications with negative impact on bladder function, such as analgesics, ACE-inhibitors, alpha-1-blocker, benzodiazepines, antihistamines, antidepressants, ...)<br>For urge incontinence: Because of side effects, therapy trial with beta-3 adrenergic agonist (mirabegron or vibegron) rather than with an antimuscarinic agent. Discontinue in the absence of clinical improvement.<br>Urge incontinence due to prostatic hyperplasia -> see "Prostatic hyperplasia"<br>Discontinuation of local estrogen therapy in peri-/postmenopausal women in the absence of clinical improvement.<br>Stress incontinence: No established pharmacotherapy	Watch out for anticholinergic side effects.<br><br>Trospium chloride (compared with placebo): NNT 9 in achieving continence, NNT 13 for clinical improvement<br>Tolterodine (compared to placebo): NNT 11 in achieving continence, NNT 10 for clinical improvement	N39	UpToDate (27.12.2020)<br>- Treatment of urinary incontinence in women<br>- Urinary incontinence in men<br>- Treatment of urgency incontinence/overactive bladder in women<br><br>PMID: 25222388<br>PMID: 29398262<br>PMID: 30288727<br>PMID: 25630399<br>PMID: 22711079<br>PMID: 31039103

ACE=Angiotensin Converting Enzyme, ACS=acute coronary syndrome, AP=angina pectoris, ARB=angiotensin receptor blocker, ARR=absolute risk reduction, AT-II=Angiotensin II, BPH= benign prostatic hyperplasia, CAD=coronary artery disease, CIPN=chemotherapy-induced peripheral neuropathy, CKD=chronic kidney disease, COPD=Chronic obstructive pulmonary disease, CPPD=Calcium Pyrophosphate Deposition, CRT=cardiac resynchronization therapy, CV=cardioversion, CVD=cardiovascular disease, DMARD=disease-modifying anti-rheumatic drug, DOAC=direct oral anticoagulant, DVT=deep vein thrombosis, EC=erythrocyte concentrate, EF=ejection fraction, ESA=erythropoietin stimulating agent, ESC=European Society of Cardiology, GERD=Gastroesophageal reflux disease, GFR=glomerular filtration rate, GI=gastrointestinal, GN=glomerulonephritis, HBV=hepatitis B virus, H.p.=Helicobacter pylori, HPT=hyperparathyroidism, HTG=Hypertriglyceridemia, IBD=inflammatory bowel disease, ICD=implantable cardioverter-defibrillator, ICS=inhaled corticosteroid, INR=International Normalized Ratio, LABA=long-acting beta-agonists, LAMA=long-acting anticholinergics, LVEF=left ventricular ejection fraction, MALT=mucosa-associated lymphoid tissue, MMSE=Mini-Mental State Examination, MRA=mineralocorticoid receptor antagonists, NNH=number needed to harm, NNT=number needed to treat, NSAID=non-steroidal anti-inflammatory drug, OD=once daily, PAD=peripheral artery disease, PAH=Pulmonary hypertension, PCP=Pneumocystis jirovecii, PCR=polymerase chain reaction, PMID=PubMed identification number, PNP=polyneuropathy, PPI=proton pump inhibitor, PTH=parathyroid hormone, RA=Rheumatoid arthritis, RR=risk reduction, SABA=short-acting beta-agonists, SAMA=short-acting anticholinergics, SBP=spontaneous bacterial peritonitis, SSRI=selective serotonin reuptake inhibitor, SSS=Sick Sinus Syndrome, TEE=transesophageal echocardiography, TIA=transient ischemic attack , TIPS=Transjugular intrahepatic portosystemic shunt, TSH=Thyroid Stimulating Hormone, VT=ventricular tachycardia, VTE=Venous thromboembolism
