# Supplementary material for: TOP-PIC: a new tool to optimize pharmacotherapy and reduce polypharmacy in patients with incurable cancer
Source: J Cancer Res Clin Oncol. 2023 Mar 6;149(10):7113–23. doi: 10.1007/s00432-023-04671-9 (PMC10374723; doi:10.1007/s00432-023-04671-9)
Supplement: Supplementary file 2 — Supplementary file2 (PDF 65 KB) [file 432_2023_4671_MOESM2_ESM.pdf]

**Table S2** Medication changes in Part 1 and Part 2 of the pilot test

| | | | | Number | % | Proportion of total Med % |
|---|---|---|---|---|---|---|
| Part1_Change category | unchanged | Part2_Change category | unchanged | 600 | 84,5 | 56,4 |
| | | | discontinued | 91 | 12,8 | 8,6 |
| | | | reduced | 5 | 0,7 | 0,5 |
| | | | increased | 2 | 0,3 | 0,2 |
| | | | replaced | 12 | 1,7 | 1,1 |
| | | | **Total** | **710** | **100,0** | **66,7** |
| | discontinued | Part2_ Change category | unchanged | 52 | 18,6 | 4,9 |
| | | | discontinued | 217 | 77,8 | 20,4 |
| | | | reduced | 7 | 2,5 | 0,7 |
| | | | replaced | 3 | 1,1 | 0,3 |
| | | | **Total** | **279** | **100,0** | **26,2** |
| | reduced | Part2_ Change category | unchanged | 11 | 37,9 | 1,0 |
| | | | discontinued | 2 | 6,9 | 0,2 |
| | | | reduced | 16 | 55,2 | 1,5 |
| | | | **Total** | **29** | **100,0** | **2,7** |
| | increased | Part2_ Change category | unchanged | 4 | 33,3 | 0,4 |
| | | | discontinued | 2 | 16,7 | 0,2 |
| | | | increased | 5 | 41,7 | 0,5 |
| | | | replaced | 1 | 8,3 | 0,1 |
| | | | **Total** | **12** | **100,0** | **1,1** |
| | replaced | Part2_ Change category | unchanged | 9 | 26,5 | 0,8 |
| | | | discontinued | 6 | 17,6 | 0,6 |
| | | | replaced | 19 | 55,9 | 1,8 |
| | | | **Total** | **34** | **100,0** | **3,2** |
